# The broccoli (*Brassica oleracea*) phloem tissue proteome

**DOI:** 10.1186/1471-2164-14-764

**Published:** 2013-11-07

**Authors:** James A Anstead, Steven D Hartson, Gary A Thompson

**Affiliations:** 1 College of Agricultural Sciences, The Pennsylvania State University, University Park, PA 16802, USA; 2 Department of Biochemistry & Molecular Biology, Oklahoma State University, Stillwater, OK 74074, USA

**Keywords:** *Brassica oleracea*, Phloem, Proteome, Sieve-elements, Protein

## Abstract

**Background:**

The transport of sugars, hormones, amino acids, proteins, sugar alcohols, and other organic compounds from the sites of synthesis to the sites of use or storage occurs through the conducting cells of the phloem. To better understand these processes a comprehensive understanding of the proteins involved is required. While a considerable amount of data has been obtained from proteomic analyses of phloem sap, this has mainly served to identify the soluble proteins that are translocated through the phloem network.

**Results:**

In order to obtain more comprehensive proteomic data from phloem tissue we developed a simple dissection procedure to isolate phloem tissue from *Brassica oleracea*. The presence of a high density of phloem sieve elements was confirmed using light microscopy and fluorescently labeled sieve element-specific antibodies. To increase the depth of the proteomic analysis for membrane bound and associated proteins, soluble proteins were extracted first and subsequent extractions were carried out using two different detergents (SDS and CHAPSO). Across all three extractions almost four hundred proteins were identified and each extraction method added to the analysis demonstrating the utility of an approach combining several extraction protocols.

**Conclusions:**

The phloem was found to be enriched in proteins associated with biotic and abiotic stress responses and structural proteins. Subsequent expression analysis identified a number of genes that appear to be expressed exclusively or at very high levels in phloem tissue, including genes that are known to express specifically in the phloem as well as novel phloem genes.

## Background

The phloem tissue of plant vascular systems forms the functional conduit for transporting photosynthates, macromolecules and other organic compounds from the sites of synthesis to the sites of use or storage. The phloem is a complex tissue composed of multiple cell types that have specific functions in translocation, structure and defense. The highly specialized conducting cells, sieve elements (SEs) are connected by perforated sieve plates to form sieve tubes; a living, functional conduit of cells that allows low resistance movement of sap throughout the plant (Oparka and Simon 2000). During development, the cytoplasmic contents of SEs are extensively restructured to provide an open and continuous lumen for translocation. Due to the developmental degradation of the nucleus, ribosomes and Golgi bodies, the SE is dependent for many of its functions on its neighboring companion cells (CCs), establishing a functional complex between the two cell types that facilitates the exchange of molecules through pore-plasmodesmatal connections. Thus, proteins are either synthesized in immature SEs or transported from CCs. The SE-CC complex is embedded within phloem parenchyma cells, and the phloem tissue can contain other specialized cells such as phloem fibers that provide structural support or cells that are involved in defense mechanisms. An example of a unique defense mechanism in brassicas involves S-cells located between the endodermis and phloem that accumulate glucosinolates and associated M-cells within the phloem that produce myrosinase, an enzyme that catalyzes the hydrolysis of glucosinolates into potent plant defense compounds [1]. There is also a growing body of evidence that the phloem transports macromolecules, including proteins and RNAs that are involved in plant defense, maintaining cellular functions and as developmental signals [2-9].

Proteomic investigations of the phloem tissue have predominantly focused on the analysis of phloem sap exudates, identifying several hundred physiologically relevant proteins and ribonucleoprotein complexes within the translocation stream. Proteome analysis of phloem sap has been conducted in oilseed rape (*Brassica napus*), hybrid poplar (*Populus trichocarpa* × *Populus deltoids)*, rice (*Oryza sativa*), pumpkin (*Cucurbita maxima*), cucumber (*Cucumis sativus*), and melon (*Cucumis melo*) [4,9-14]. Highly represented among these studies are proteins involved in redox regulation, defense and stress responses, and calcium regulation. Changes in the phloem sap proteome have also been investigated in response to external stimuli. In poplar, leaf wounding led to the accumulation of two proteins; SP1 and a pathogen-related (PR-5) family protein [12]. Similarly, melon plants infected with Cucumber mosaic virus accumulated additional plant defense proteins in the phloem sap [11].

The vasculature is embedded within ground tissue, especially in stems, making it difficult to isolate large amounts of highly enriched phloem tissue required for proteomic analysis. In contrast to phloem sap proteins, there is much less information available about proteins present throughout the phloem tissue. Such data could reveal information about the presence and function of membrane-associated or complexed proteins within the SEs or proteins present in other cell types within the phloem tissue. Laser-microdissected vascular bundles have been successfully used for proteomic analysis in *Arabidopsis*[15]. Despite the low volume of tissue recovered by this technique, 49 proteins were identified from 5000 micro-dissected vascular bundles with comparative analysis of tissue sections with and without vascular bundles identifying 17 vascular bundle proteins [15]. In another study, nano-LC-MS/MS was used to identify 56 proteins from pooled *Arabidopsis* S-cells sampled using glass capillaries [16], these included proteins that formed the biosynthesis machinery for methionine and hence glucosinolates as well as high amounts of TGG1 and TGG2. This indicated that in *Arabidopsis* myrosinases and glucosinolates can be localized in the same cells, presumably in different compartments. Isolated strands of phloem tissue from celery petioles (*Apium graveolens*) have also been used for transcriptomic studies, revealing mRNAs encoding tissue-specific expression patterns for several major classes of phloem proteins. Genes were identified encoding a number of structural proteins including phloem lectins, various cell wall associated genes and cytoskeletal proteins as well as proteins involved in metal homeostasis, stress responses, including genes associated with JA synthesis and degradation or turnover of proteins [17].

Other approaches have focused on individual proteins that are associated with the unique physiological functions or structures in the phloem. For instance, sieve element occlusion and sieve element occlusion related proteins (SEO and SEOR) have been identified and localized in a number of systems including *Arabidopsis*[18-20]. Enzymes involved in sugar metabolism and transport were also found to be phloem-specific. Two sucrose synthase genes (SUS5 and SUS6) are expressed exclusively in the phloem [21,22]. Sucrose transporters are also highly expressed in phloem tissue and phloem-specific transporters have been identified in many different plant species [23]. In *Arabidopsis* other research has focused on phloem-associated lipid binding proteins [2] and enzymes involved in the Yang cycle [24].

In this study, a simple technique was used to isolate large quantities of phloem-enriched tissue to study the phloem proteome of broccoli (*Brassica oleracea*). The vascular architecture in the stem of broccoli is composed of large, easily identifiable phloem strands that can be physically separated from the surrounding tissues, particularly the xylem and epidermis. Differential extraction methods combined with LC-MS/MS revealed different classes of soluble and membrane-associated proteins. Because *Brassica oleracea* and *Arabidopsis thaliana* are both members of the family Brassicaceae, protein identification was facilitated by the availability of the well-annotated *Arabidopsis* genome allowing a more in-depth functional analysis.

## Methods

### Tissue dissection

Stems from commercially grown broccoli crowns were scored with a double-edged razor blade near the base into cylinder-like sections ~3-5 cm wide at a depth of ~1-2 mm. A vertical slice was made to expose the cambium, and the exterior layer composed mostly of the epidermis was peeled off using fine forceps under a binocular microscope. The majority of the phloem tissue was removed with the epidermal peel, leaving behind the xylem tissues. Strands of phloem-enriched tissue were prepared by peeling phloem fibers from the epidermal peel with a probe under the binocular microscope. Control tissue, containing both pith and xylem tissue, but no phloem, was extracted from the same stem sections using a 2.5 cm core borer. Dissected tissues were flash frozen in liquid nitrogen, weighed and stored at -80°C.

### Immunolocalization

Two different phloem-specific monoclonal antibodies, RS6 and RS32, were used to visualize SEs within the excised phloem-enriched tissue. The R6 antibody readily cross-reacts with the protein antigen in *B. oleracea* that is homologous to the *Arabidopsis* Sieve Element-specific Early Nodulin (SE-ENOD) encoded by At3g20570 [25]. The RS32 antigen has been designated as p35 for an unidentified 35 kDA protein that localizes to SEs in *Brassica* and *Arabidopsis* (Sjolund R – pers. Comm.).

Excised phloem-enriched tissues from *B. oleracea* were washed twice in 10 mM PBS and incubated for 30 minutes in PBS with 3% non-fat dry milk (blocking buffer). Strands were then washed twice with PBS and incubated for 45 minutes with each monoclonal antibody in blocking buffer (1:100). After incubation with primary antibody, the strands were washed three times with PBS and then incubated in PBS with ALEXA 488 nm fluorescently tagged secondary goat anti-mouse antibody (Invitrogen, Carlsbad, CA) (1:250). The labeled tissues were washed twice with PBS and once with nanopure water and observed under a Nikon E600 epifluorescence microscope with an excitation wavelength of 490 nm and an emission wavelength of 512 nm.

### Protein extraction

Two grams of phloem-enriched tissue were ground in liquid nitrogen with a mortar and pestle and extracted with 4 ml of soluble protein extraction buffer (10 mM Tris pH 7.2, 10 mM EGTA, 150 mM NaCl, 10 mM KCl, 1% Sigma plant protease inhibitor cocktail, 20 mM dithiothreitol). The tissue was incubated in the soluble extraction buffer for one hour on a rocking platform at 4°C. Soluble proteins were removed following centrifugation at 17,000 rpm for 25 minutes in JA 20 rotor in Avanti J-E centrifuge (Beckman Coulter). Tissues were resuspended in 4 ml of either CHAPSO (10 mM Tris pH 7.2, 10 mM EGTA, 150 mM NaCl, 10 mM KCl, 1% Sigma plant protease inhibitor cocktail, 20 mM dithiothreitol) or SDS buffer (4% SDS, 125 mM Tris–HCl pH 7.2, 150 mM NaCl, 10 mM KCl, 50 mM dithiothreitol, 1% Sigma plant protease inhibitor cocktail) and incubated at room temperature for 1 hour on a rocking platform. Four ml of the supernatant containing total membrane proteins were collected following centrifugation as described above. Protein concentrations were determined with the RCDC protein assay kit (Bio-Rad, catalog no.500-0119), which is compatible with CHAPS and SDS. Aliquots of the aqueous and detergent extracted protein fractions were flash frozen in liquid nitrogen and stored at -80°C.

Prior to mass spectrometry proteins were concentrated using TCA-acetone that removed components such as SDS that interfere with mass spectrometry. Protein samples were dissolved in 8 M urea, 100 mM TrisHCL pH = 8.5, 5 mM tris(2-carboxyethyl)phosphine and denatured at room temperature for 20 min. After incubation, 1/20th volume of 200 mM iodoacetamide was added, and the alkylation was allowed to proceed for 15 min in the dark at room temperature. The sample was then diluted with four volumes of 100 mM TrisHCl and digested with 4 μg/ml sequencing grade trypsin (Promega V511C) overnight at 37°C. Digested samples were acidified to 1% formic acid, purified by reversed-phase chromatography using C18 affinity media (OMIX tips from Agilent), and three analytical replicates analyzed by mass spectrometry.

### LC-MS/MS

Samples were analyzed on a hybrid LTQ-Orbitrap mass spectrometer (Thermo Fisher Scientific) coupled to a New Objectives PV-550 nanoelectrospray ion source and an Eksigent NanoLC-2D chromatography system. Peptides were analyzed by trapping on a 2.5 cm ProteoPrepII pre-column (New Objective) and analytical separation on a 75 μm ID fused silica column packed in house with 10-cm of Magic C18 AQ, terminated with an integral fused silica emitter pulled in house. Peptides were eluted using a 5-40% ACN/0.1% formic acid gradient performed over 40 min at a flow rate of 300 nL/min.

During each one-second full-range FT-MS scan (nominal resolution of 60,000 FWHM, 300 to 2000 m/z), the three most intense ions were analyzed via MS/MS in the linear ion trap. MS/MS settings used a trigger threshold of 8,000 counts, monoisotopic precursor selection (MIPS), and rejection of parent ions that had unassigned charge states, were previously identified as contaminants on blank gradient runs, or were previously selected for MS/MS (data dependent acquisition using a dynamic exclusion for 150% of the observed chromatographic peak width).

### Data analysis

Centroided ion masses were extracted using the extract_msn.exe utility from Bioworks 3.3.1 and were used for database searching with Mascot v2.2.04 (Matrix Science) and X! Tandem v2007.01.01.1 (http://www.thegpm.org). Searches were conducted using the following search parameters: parent ion mass tolerance 15 ppm; fragment ion mass tolerance 0.8 Da; up to one missed trypsin cleavage; and variable modifications pyroglutamate cyclization of glutamine, oxidation of methionine, acylamide or iodacetamide adducts of cysteine, formylation or acetylation of the protein N terminus. Mass spectra were searched against a local copy of the NCBI compiled on 03/28/10, and filtered to contain only either Viriplantae or *A. thaliana* sequences, as well as reversed sequence decoys. Peptide and protein identifications were validated using Scaffold v2.2.00 (Proteome Software) and the Peptide Prophet algorithm [26]. Probability thresholds were greater than 95% probability for protein identifications, based upon at least 2 peptides identified with 80% certainty. Proteins that contained similar peptides and could not be differentiated based on MS/MS analysis alone were grouped to satisfy the principles of parsimony.

### Semi-quantitative PCR

Total RNA was extracted from both the phloem-enriched and control tissue using the Trizol method and reverse transcribed using SuperScript II according to the manufacturer’s instructions. Primers were designed to amplify partial, intron spanning sections of each Arabidopsis gene identified using VectorNTI (Invitrogen). Primers which successfully amplified are listed in Additional file 1: Table S1. Gene fragments were amplified by PCR (95°C for 7 min, followed by 40 cycles of 95°C for 15 seconds, 55°C for 15 seconds, 72°C for 90 seconds, followed by 72°C for 7 minutes). Products were separated by agarose gel electrophoresis and visualized with ethidium bromide.

## Results

### Phloem-enriched tissue extraction

**Figure 1:**
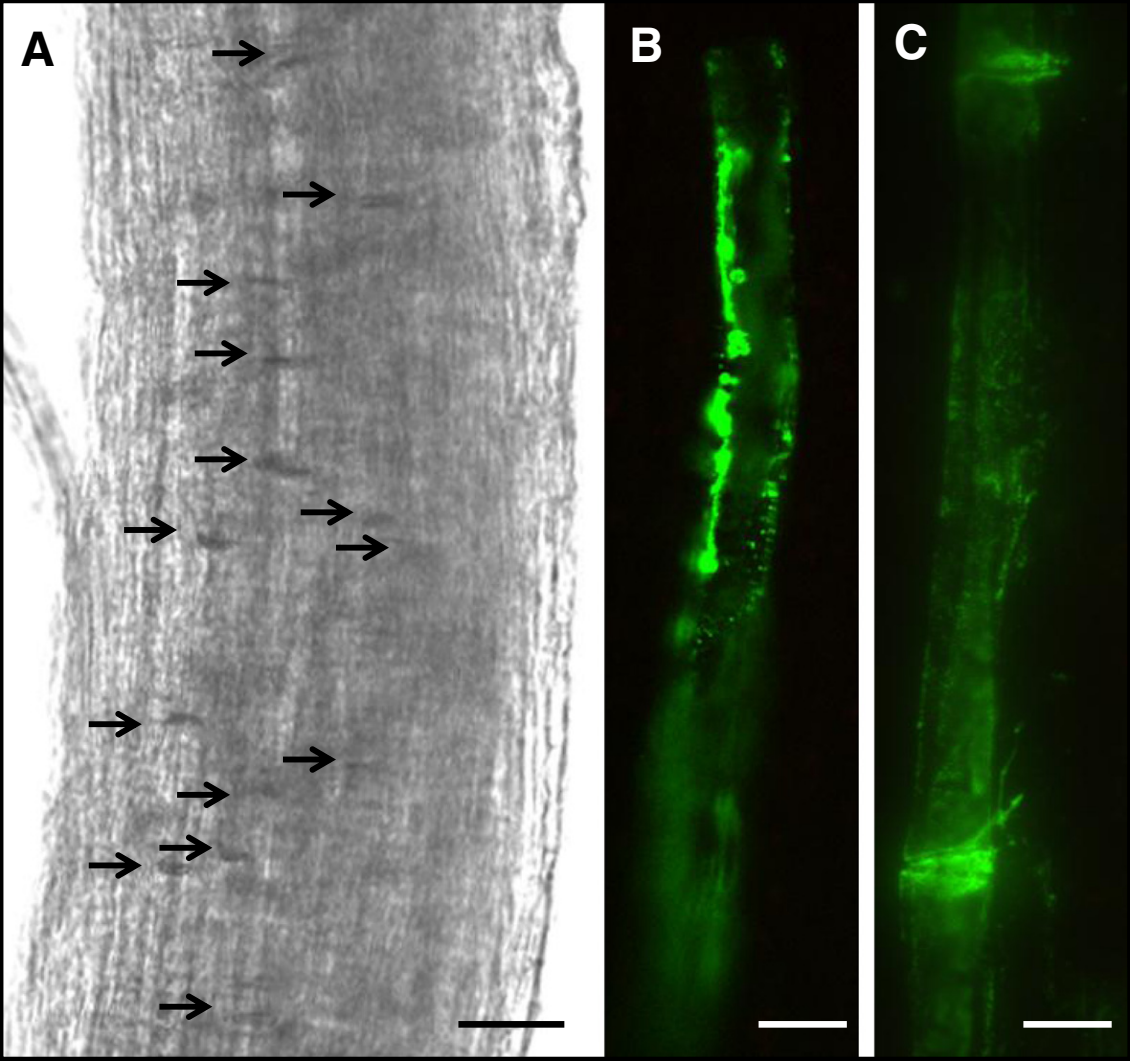
**Phloem-enriched strands isolated from *Brassica oleracea. *(A)** Brightfield micrograph of the phloem-enriched strands showing numerous sieve plates (arrows) (bar = 200 μm). **(B-C)** Immunolocalization of sieve elements within the phloem-enriched strands, containing phloem-specific protein antigens for monoclonal antibodies that recognize the **(B)** SE-ENOD and **(C)** SE-specific p35 protein (bar = 100 μm).

The large stems of broccoli crowns proved to be a useful source to isolate strands of phloem-enriched tissue. The outer layer composed mostly of epidermis and adjacent cells was easily peeled from the stem. These sections contained vertical files of phloem tissue that had separated at the cambium from the xylem. Phloem-enriched strands were readily separated from the peeled outer layer containing the epidermis. Large numbers of sieve elements with their connecting sieve plates in the isolated strands could be observed by light microscopy (Figure 1). The presence of previously characterized SE-specific proteins SE-ENOD (Figure 1B) and p35 (Figure 1C), respectively, in SEs within the excised tissue was confirmed by immunolocalization experiments with RS6 and RS32 monoclonal antibodies.

### Protein identifications

**Figure 2:**
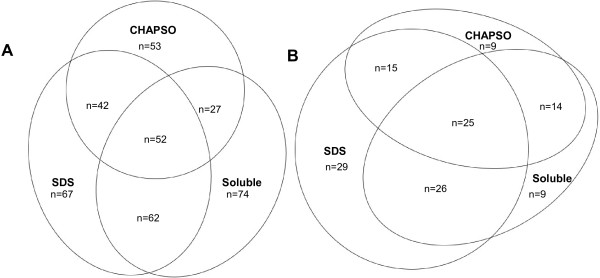
**Venn diagrams illustrating phloem proteins identified in phloem-enriched tissues. A**. The total number of proteins and **B**. The number of previously published phloem proteins identified in phloem-enriched tissues using different protein extraction protocols.

Three extraction protocols were used to isolate protein from the phloem-enriched strands. An aqueous soluble fraction was first extracted from the tissues, which were subsequently extracted with either a non-denaturing (CHAPSO) or denaturing (SDS) detergent-containing buffer, all three samples were then analyzed using LC MS/MS. Peptide identities were carefully validated using Scaffold’s (v2.2.00) Mascot, Xtandem!, and Peptide Prophet modules (Additional file 2: Table S2). Protein identifications were validated using Scaffold and the Peptide Prophet algorithm [26] (Additional file 3: Table S3). A total of 379 total proteins were identified that matched *Arabidopsis* genome data from the combination of all three extraction protocols and all LC-MS/MS runs (Additional file 3: Table S3). False discovery rates calculated by using the Protein Prophet algorithm were 0.075 for spectra and 0.002 for proteins. Proteins ranged from the most common mitochondrial ATP synthase subunit alpha to the p23 co-chaperone which was only detected once. Overall, the highest number of proteins (223) was detected in the SDS fraction and the largest number of unique proteins in the soluble (74) (Figure 2). A large number of unique proteins were also identified in the SDS fraction (67) and CHAPSO (53) fraction. A total of 127 proteins from all three extraction protocols were previously identified from proteomic analyses of phloem in several species with the SDS fraction containing the most previously identified proteins (95 out of 127) (Figure 2 and Additional file 3: Table S3).

### Functional analysis

**Figure 3:**
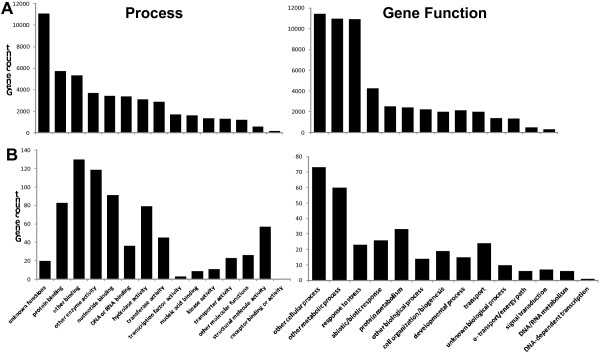
**Functional classification of phloem proteins compared to the Arabidopsis genome. ***Arabidopsis* whole genome **(A)** compared to identified phloem protein genes **(B)** using GO slim annotations, analyzed by gene count and separated into Go biological process (process) and GO molecular functions (gene function).

The biological functions and processes of the *B. oleracea* phloem-enriched strand proteins were analyzed using the currently available gene ontology (GO) annotations in *Arabidopsis* (Figure 3 and Additional file 4: Table S4). Though not a definitive analysis of function GO annotations can provide an indication of gross changes in function between tissues. Comparing GO slim annotation categories of proteins with known gene functions between the *B. oleracea* phloem proteome and the entire *Arabidopsis* genome, the largest enrichment was in GO annotations in structural molecule activity (6% versus 1%), nucleotide binding (11% versus 7%), other binding (18% versus 11%) hydrolase (13% versus 8%) and other enzyme activity (21% versus 9%). Conversely there was a paucity of annotations in nucleic acid binding (1% versus 3%), kinase activity (2% versus 6%) and transferase activity (7% versus 11%). Comparing GO slim annotation categories for proteins involved in known biological processes, the greatest differences were in responses to abiotic or biotic stimulus (9% versus 5%) and stress (10% versus 5%). In both the gene function and biological process annotation, there was a large decrease in genes assigned as unknowns in the phloem proteome when compared to the *Arabidopsis* genome, probably as a result of the inclusion of non-protein coding genes within the genome.

**Figure 4:**
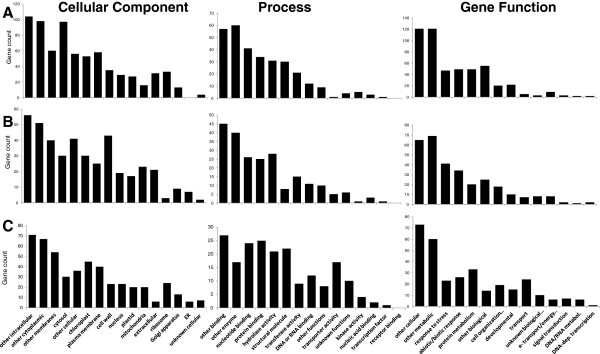
**A comparison of the classification of phloem proteins extracted using different methodologies.** Functional classification of *Brassica oleracea* phloem proteins using *Arabidopsis* GO slim annotations genes analyzed by gene count and separated into Go Cellular components (cellular component) biological process (process) and GO molecular functions (gene function). **A**: soluble fraction, **B**: Chapso fraction, **C**: SDS fraction.

A comparison was also made between the gene ontology annotations for proteins identified from each of the three extraction protocols (Figure 4 and Additional file 5: Table S5). As expected there was a higher proportion of cytosolic proteins in the soluble fraction (soluble 12% of annotations = 97 proteins; CHAPSO 6% of annotations = 30 proteins; SDS 5% of annotations = 30 proteins) (Figure 4A). The CHAPSO fraction contained the highest proportion of cell wall associated proteins (CHAPSO 9% of annotations = 43 proteins; SDS 3% of annotations = 23 proteins; soluble 3% of annotations = 35 proteins). The largest and most obvious difference among the extraction protocols involved proteins with transporter activity. The SDS extracted fraction contained many more proteins with transporter activity (14% of annotations = 39 proteins) than either the CHAPSO (2% of annotations = 5 proteins) or the soluble buffer (< 1% of annotations = 1 protein). This was supported by biological processes annotations where 52 proteins (10% of the annotations) in the SDS sample indicated involvement in transport versus only 5 proteins (1% of the annotations) and 12 proteins (2% of the annotations) in the soluble and CHAPSO fractions, respectively. The SDS extraction also outperformed the CHAPSO for structural molecules with 8% of annotations and 22 proteins versus 3% of annotations and 8 proteins.

### Gene expression

**Figure 5:**
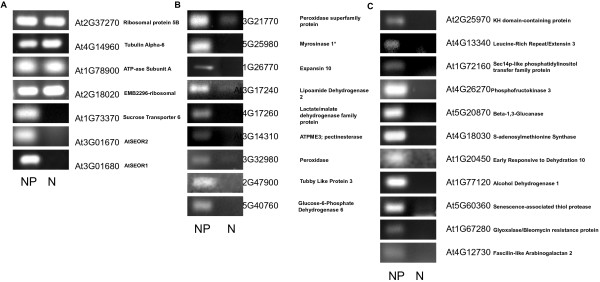
**Gene expression in *B. oleracea *phloem-enriched strands control tissue.** Semi-quantitative RT-PCR analysis of gene expression in *B. oleracea* phloem-enriched strands (P) and non-phloem containing control tissue (NP). **A**. Controls: four genes encoding housekeeping genes with expression in both phloem and non-phloem tissues and three phloem-specific genes encoding sucrose synthase 6, SEOR1, and SEOR2. **B**. The expression of genes encoding proteins previously identified as phloem-specific that were identified in the *B. oleracea* proteome. **C**. The expression of genes encoding proteins identified in the *B. oleracea* proteome that were not previously identified as phloem-specific proteins. *Myrosinase 1 primers also amplified myrosinase 2 (At4G26000).

Semi-quantitative RT-PCR was used to examine whether the genes encoding the 377 proteins identified in the phloem were expressed in the excised phloem-enriched strands. To determine whether any of these genes showed evidence of high or phloem-specific expression, expression in the phloem-enriched strands was compared with pith (non-phloem) tissue isolated from stems. Two sets of control genes that are known to be either ubiquitously expressed or expressed only in the sieve elements in *Arabidopsis* were used to confirm the absence of phloem in the phloem-enriched sample and control for expression levels (Figure 5). These genes confirmed the absence of sieve elements in the pith tissue sample. From the 377 primer pairs designed, 166 gave amplification products detectable by ethidium bromide electrophoresis (Additional file 6: Figure S1). There were no genes that showed expression in the control tissue alone although several had comparatively much higher expression than in the phloem, perhaps due a lower frequency of specific cell types such as parenchyma in the phloem tissue. For example, At3g50820 which encodes a photosystem II subunit and At5G65760 which encodes a serine carboxypeptidase exhibited comparatively lower expression in the phloem tissue (Additional file 4: Table S4). In addition to the three control genes 20 other genes showed enhanced or possible phloem-specific expression (Figure 5).

## Discussion

### Phloem dissection and protein extraction

One of the most significant hurdles to successful proteomic analysis of plant phloem tissue is obtaining sufficient amounts of phloem tissue for analysis. Many studies have focused on phloem exudates, mostly from cucurbits, where large volumes of sap can be obtained. The most significant drawback to this approach is that it limits the analysis to proteins that are soluble in the sap, ostensibly being translocated in the phloem. Furthermore, questions have been raised concerning the broad applicability of proteomic data obtained from cucurbit phloem sap due to their unusual phloem anatomy. Cucurbits possess two ontogenetically distinct phloem systems; the fascicular phloem of the vascular bundle, which might be considered homologous to the phloem in other plant families and the extra-fascicular phloem located at the periphery of the vascular bundles and scattered throughout the stem. There is mounting evidence that these phloem systems are functionally distinct and that the extra-fascicular phloem contributes the majority of the sap used in proteomic and transcriptomic analyses [27]. Proteomic data from phloem sap in other plant systems, such as rice and brassicas, that do not readily exude large volumes of sap have been obtained, but the analysis is still limited to sap-soluble proteins [13,28]. To reveal the broader phloem proteome containing soluble, membrane-associated and integral membrane proteins, a relatively simple protocol was used to dissect phloem-enriched strands from broccoli (*B. oleracea*). Light microscopy and immunolocalization analysis with well-defined phloem-specific monoclonal antibodies demonstrated that phloem-enriched strips contain abundant sieve elements (Figure 1). Most of the proteins identified can be easily understood as phloem constituents, and obvious contaminants were not apparent. This was also borne out by the high level of expression of phloem-specific genes in the phloem-enriched tissues when compared to stem pith tissue that lacked phloem marker gene expression (Figure 5). Whilst the phloem strands are highly enriched in sieve elements and other phloem cells, it should be noted that non-phloem cells from surrounding ground tissue could be included in the analysis.

### Extraction methodologies

Several approaches were combined to increase depth of sequencing for membrane and membrane-associated proteins from phloem-enriched tissues. Firstly soluble proteins were removed using a simple salt-wash and analyzed separately and secondly different detergents, CHAPSO and SDS, were used to extract a wider range of protein classes from the remaining tissue. Creating differentially extracted pools of proteins revealed differences in both the amount and types of protein that could be identified. Small differences in the number of proteins were obtained using each approach; however, all three fractions contained unique proteins (Figure 2), indicating that a combined approach using several extraction protocols provided the deepest data set. The SDS fraction contained many more proteins involved in transport and transport activity (Figure 4). These included membrane proteins such as Shepherd (SHDP) ATP/ADP carrier 1 (AAC1) and V-type proton ATPase 16 kDa proteolipid subunit c1 (AVP1), which were only identified in the SDS extracted fraction. Interestingly, of the three sucrose synthases identified, two (SUS1 and 4) were only identified in the soluble fraction and the other (SUS6) was only identified in the SDS fraction. This agrees with the GO annotation details for these genes as SUS6 is reported to be located within the chloroplast, whereas SUS1 and 4 are cytosolic proteins [29,30]. Cell wall proteins were optimally extracted with CHAPSO in the buffer, and leucine rich repeats proteins and peroxidases were only identified in this fraction.

### Identified proteins

LC MS/MS analysis identified approximately four hundred different proteins, considerably more than in previously published datasets. There was however considerable overlap with previously published data sets that identified proteins predominantly from phloem exudates, but also from micro-dissected phloem tissues and S-cells (Figure 2 and Additional file 2: Table S2). Twenty-one of the 49 proteins identified in micro-dissected vascular bundles from *Arabidopsis* were also found in the broccoli phloem-enriched tissues [15]. Similarly, 27 of the 56 proteins identified from *Arabidopsis* S-cells were found in the broccoli dataset [16]. The absence of some identified proteins in the *Brassica oleracea* proteome are likely due to divergence between *Brassica oleracea* and *Arabidopsis*. The low frequency of S-cells within phloem tissue also means a whole phloem proteome will have less depth for S-cell proteins. However overall this simple dissection protocol produced a deeper proteome than previous attempts and was particularly rich in membrane and membrane associated proteins.

### Gene processes and biological function

Identifying phloem proteins using the *Arabidopsis* genome allowed comparisons to be made between GO annotations associated with phloem proteins and those associated with the whole *Arabidopsis* genome. At a broad level this shows how the specialized function of the phloem tissue differs from the rest of the plant. For instance the largest differences between the whole plant and phloem-enriched tissues in biological processes were proteins involved in response to biotic and abiotic stimulus and stress and in gene function were in structural molecule activity. Additional transcriptional analysis showed 20 *Brassica* homologs to *Arabidopsis* genes were found to have very high expression in the phloem-enriched tissue when compared to the control stem pith tissue (Figure 5). Little has been published about some of these proteins, but many of them are encoded by members of large gene families suggesting that these genes represent tissue-specific members within the gene family.

### Biotic and abiotic stimuli and stress

The enhanced presence of proteins involved in responses to biotic and abiotic stimuli and stress reflect a similar enrichment of stress-regulated genes found in the transcriptomic analysis of celery phloem [17]. This is perhaps not surprising as specialist insect herbivores such as aphids and whiteflies feed exclusively in this tissue. In response to this specialized herbivory, plants have evolved a range of induced responses including activation of jasmonic acid, salicylic acid and ethylene defense pathways, the production of pathogenesis-related (PR) proteins, proteins involved in reactive oxygen species processes and calcium signaling [7,31-35]. Proteins involved in generalized plant defenses are also present in the phloem, including a large number of protease inhibitors [7] and the glucosinolate-based defense pathway [1]. *Arabidopsis* possesses six myrosinase genes encoding proteins which are expressed in guard and phloem cells and degrade glucosinolates to produce herbivory deterring compounds [36]. In this study, two myrosinases proteins (TGG1 and TGG2) were identified in the phloem proteome as well as a number of other proteins involved in the production of glucosinolate defense compounds, such as myrosinase-binding protein-like protein (At1G52030) and a myrosinase-associated protein (At1G54000). Myrosinase expression was high in the phloem enriched tissue and not detectable in the control tissue (Figure 5); given that the control tissue used in this experiment contained no guard cells, expression in the phloem tissue alone was to be expected. Viral defense proteins have also been previously reported from phloem tissue; the RTM (Restricted TEV Movement) family have been shown to restrict the long distance movement of potyviruses in the phloem [37,38]. A RTM2 (AT5G04890) homolog was identified in the *B. oleracea* in our phloem proteome.

β-1, 3-glucanase is involved in many different processes, including pathogen, stress and hormone responses as well as developmental processes. A putative (At3G55430) and a confirmed β-1, 3-glucanases (At5G20870) were identified in the phloem proteome. At5G20870 was shown to have high comparative expression in the phloem (PCR amplification failed for At3G55430). Expression analysis of this family indicates At3G55430 is up-regulated in reponse to fungal pathogen infestations and that At5G20870 may be involved in developmental processes, although this is by no means certain [39]. At5G60360.1 is a vacuolar protein [40] that is responsive to biotic stress and ethylene [41]. The identification and comparatively higher expression of genes responsive to stress is likely related to the role of the phloem in response to abiotic stresses such as drought and salinity which perturb the phloem transport system.

The phloem is also responsive to a variety of abiotic stresses. Water plays an important role in the long-distance transport of compounds through phloem sieve tubes, thus, it was not surprising to find a number of dehydration-related proteins such as dehydrin, RD (Responsive to Dehydration) and ERD (Early-responsive to dehydration) proteins. Other proteins involved in abiotic stress responses, such as cold-regulated and heat-shock proteins were also identified.

These include glucose-6-phosphate dehydrogenase 6 (G6PD6) and the lactate/malate dehydrogenase family protein (At4G17260), both previously identified in pumpkin phloem exudates [10]. G6PD6 provides NADPH for redox regulation in response to ROS stress that when activated by ASK-alpha (Glycogen Synthase Kinase 3) reduces ROS levels induced by salt-stress, increasing salt-tolerance [42]. The lactate/malate dehydrogenase family protein (At4G17260) is also reported to be responsive to salt-stress and ABA [43,44]. ERD10 (At1G20450) is responsive to dehydration [45] and cold-stress; and ERD10 loss of function *Arabidopsis* mutants show reduced tolerance to cold and drought stress and low seed germination [46]. ERD10 also undergoes oxidation of specific methionine residues in response to stress, thus regulating cellular responses [47]. The Glyoxalase/Bleomycin resistance protein encoded by At1g67280 is expressed in all developmental and induced by abiotic stresses such as salinity, drought, cold, and heat in shoot and root tissues [48]. Similarly ADH1 is expressed in multiple plant tissues and is highly responsive to multiple abiotic stresses including drought and salt stress [43,49].

Tubby-like proteins are involved in plant-stress signaling and are integrated into a number of plant-stress response pathways [50]. TLP3 is believed to act as a stress-responsive plasma membrane-tethered transcription factor [51] and in plants acts in both stress responses and reactive oxygen species (ROS) signaling [52]. GUS promoter expression analysis showed Tubby-like Protein 3 to be highly, but not exclusively, expressed in vascular tissue [52].

### Structural proteins

Large numbers of proteins associated with structural molecule activity were also present in the dataset. The majority of these were ribosomal proteins, but there were also several members of the actin (Actin2 & 7) and profilin families (Profilin 1 & 5) as well as a six members of the tubulin family (Tubulin-Alpha 3 & 6, and Tubulin-Beta 1,2 4 & 5). Historically, phloem sieve elements were believed to lack a conventional cytoskeleton; however actin and profilin proteins have been previously reported in phloem exudates in a number of plant species [9,10,13,53] and recent evidence has unequivocably shown that SE’s contain a fully developed actin network [54]. In addition to actin and profilin, an expansin (At1G26770) and clathrin (At3G11130) were identified in the *B. oleracea* proteome. Expansin 10 is a member of a large-multi-gene family whose members tend towards tissue or cell specific expression patterns, regulating cell wall enlargement in growing cells by a process that appears to induce pH-dependent wall extension and stress relaxation [55,56]. Expansin 10 has been previously reported to show a phloem-cambium expression bias and these data support a possible phloem-specific role for this protein [57].

Pectin methylesterases are another large family of enzymes involved in cell-wall restructuring. Many members of this family are expressed in a tissue-specific manner during developmental periods such as stem elongation [58] and fruit ripening [59]. Promoter analysis of ATPME3 expression shows highly specific expression in phloem tissue [60] in concordance with the data presented here. Interestingly ATPME3 acts as a susceptibility factor and is required for infection by necrotrophic pathogens [61], indicating a possible route for phloem cell wall penetration by these pathogens.

Additional highly expressed cell wall associated proteins were also identified, including two peroxidases (At3G21770, At3G32980) that had been previously identified from phloem samples [62], a Leucine Rich Repeat cell wall protein (At4G13340) and a fasciclin-like arabinogalactan (FLA2, At4G12730). Little is known about the specific biological functions of these proteins, although fasciclin-like arabinogalactans are believed to play a role in secondary plant cell wall biosynthesis and other members of the family show tissue-specific patterns of expression [63]. The presence of these specific cell wall proteins is likely associated with the unique structure of phloem cells, particularly SEs and CCs. The same may be true of PFK3 (phosphofructokinase 3, At4G26270) that was also highly expressed in phloem tissue. PFK3 regulated by HDA18 HISTONE DEACETYLASE 18 and involved in cell patterning and fate [64] and could play a role in phloem differentiation.

### Other proteins

Phloem is believed to be the major transport route for sulphur in plants and considerable data exists on long-distance transport of sulfur-containing compounds in the phloem. The enzyme S-adenosylmethionine synthase (SAM-2) that generates S-adenosylmethionine from methionine and ATP was identified in the *B. oleracea* phloem-enriched proteome. A related methionine S-methyltransferase that catalyses the step of the methionine synthesis pathway producing S-methylmethionine (SMM) from S-adenosylmethionine has been found in phloem exudate collected from aphid stylectomies in wheat [65]. Both these enzymes are required to convert methionine to SMM, which is believed to play a major role in sulphur transport in phloem tissue [65,66].

Proteins were identified with less defined roles in phloem biology. The amino acid sequence of the KH binding domain protein (At2G25970) indicates that this is a putative RNA binding protein. While additional information for this particular protein is lacking, long distance RNA trafficking is believed to occur in the phloem with the assistance of a number of RNA binding proteins [6,67]. Patellin-3 (PATL-3) was also found to be highly expressed in *B. oleracea* phloem-enriched tissue. Patellins are a six member family of membrane proteins in *Arabidopsis,* PATL1, the best characterized patellin, is a phosphoinositide-binding protein that localizes to the expanding and maturing cell plate [68], however little information is available regarding PATL3 and its possible role in phloem physiology remains to be elucidated.

## Conclusions

A simple dissection technique was described that generated large quantities of phloem tissue from *Brassica oleracea*. Analyses using phloem specific antibodies and proteomic analyses indicated it was highly-enriched for phloem tissue. Soluble and membrane associated proteins were extracted using several different techniques and analyzed using LC MS/MS to create a deep proteome data set. A total of 377 proteins were identified and analyzed using Gene Ontology terms. When compared to the whole Arabidopsis genome the *B. oleracea* phloem was enriched for structural proteins and proteins related to biotic and abiotic stimuli and stress. Subsequent transcriptional analyses identified a smaller sub-set of genes that are highly or exclusively transcribed in phloem tissue and their functional significance is discussed.

## Availability of supporting data

Mass spec data has been deposited at Peptide Atlas (PASS00331).

## Competing interests

The authors declare that they have no competing interests.

## Authors’ contributions

The experiments were conceived and designed by JAA and GAT. JAA and SDH performed the experiments and analyzed the data. The manuscript was written by JAA and GAT. All authors read and approved the final manuscript.

## Supplementary Material

Additional file 1: Table S1 Primer sequences used to amplify gene fragments from Arabidopsis genes identified from phloem protein sequences. Click here for file

Additional file 2: Table S2 Peptides identified by LC-MS/MS from three different *B. Oleracea* phloem extractions and their quality statistics. Click here for file

Additional file 3: Table S3 Proteins identified by matching peptide sequences against the Arabidopsis genome, including spectrum counts for each extraction methodology and whether the protein has previously been identified in the phloem. (*indicates family ambiguity) [69-73]. Click here for file

Additional file 4: Table S4 Functional classification of phloem proteins compared to the Arabidopsis whole genome using GO slim annotations, analyzed by the proportion of annotation counts and separated into GO molecular functions and Go biological process expressed in number of proteins and percentages of the total number of proteins identified. Click here for file

Additional file 5: Table S5 Functional classification of phloem proteins by extraction process (soluble fraction, Chapso fraction or SDS fraction) analyzed by the proportion of annotation counts and separated into GO cellular components, GO molecular functions and Go biological process expressed in number of proteins and percentages of the total number of proteins identified. Click here for file

Additional file 6: Figure S1 Semi-quantitative RT-PCR analysis of gene expression in *B. oleracea* phloem-enriched strands (P) and non-phloem containing control tissue (NP). Click here for file

## References

[B1] Bones AM Rossiter JT The myrosinase-glucosinolate system, its organisation and biochemistry Physiol Plant 1996 97 1 194 208 10.1111/j.1399-3054.1996.tb00497.x

[B2] Guelette BS Benning UF Hoffmann-Benning S Identification of lipids and lipid-binding proteins in phloem exudates from Arabidopsis thaliana J Exp Bot 2012 63 10 3603 3616 10.1093/jxb/ers028 22442409 PMC3388829

[B3] Zhang S Kragler F Unspecific inhibition of translation by phloem RNAs FEBS J 2007 274 129 129

[B4] Aki T Shigyo M Nakano R Yoneyama T Yanagisawa S Nano scale proteomics revealed the presence of regulatory proteins including three FT-Like proteins in phloem and xylem saps from rice Plant Cell Physiol 2008 49 5 767 790 10.1093/pcp/pcn049 18372294

[B5] Buhtz A Springer F Chappell L Baulcombe DC Kehr J Identification and characterization of small RNAs from the phloem of *Brassica napus* Plant J 2008 53 5 739 749 10.1111/j.1365-313X.2007.03368.x 18005229

[B6] Gomez G Torres H Pallas V Identification of translocatable RNA-binding phloem proteins from melon, potential components of the long-distance RNA transport system Plant J 2005 41 1 107 116 15610353 10.1111/j.1365-313X.2004.02278.x

[B7] Kehr J Phloem sap proteins: their identities and potential roles in the interaction between plants and phloem-feeding insects J Exp Bot 2006 57 4 767 774 10.1093/jxb/erj087 16495410

[B8] Kehr J Buhtz A Long distance transport and movement of RNA through the phloem J Exp Bot 2008 59 1 85 92 17905731 10.1093/jxb/erm176

[B9] Walz C Giavalisco P Schad M Juenger M Klose J Kehr J Proteomics of curcurbit phloem exudate reveals a network of defence proteins Phytochemistry 2004 65 12 1795 1804 10.1016/j.phytochem.2004.04.006 15276438

[B10] Lin M-K Lee Y-J Lough TJ Phinney BS Lucas WJ Analysis of the pumpkin phloem proteome provides insights into angiosperm sieve tube function Mol Cell Proteomics 2009 8 2 343 356 18936055 10.1074/mcp.M800420-MCP200

[B11] Malter D Wolf S Melon phloem-sap proteome: developmental control and response to viral infection Protoplasma 2011 248 1 217 224 10.1007/s00709-010-0215-8 20924770

[B12] Dafoe NJ Zamani A Ekramoddoullah AKM Lippert D Bohlmann J Constabel CP Analysis of the poplar phloem proteome and its response to leaf wounding J Proteome Res 2009 8 5 2341 2350 10.1021/pr800968r 19245218

[B13] Giavalisco P Kapitza K Kolasa A Buhtz A Kehr J Towards the proteome of *Brassica napus* phloem sap Proteomics 2006 6 3 896 909 10.1002/pmic.200500155 16400686

[B14] Cho WK Chen XY Rim Y Chu H Kim S Kim SW Park ZY Kim JY Proteome study of the phloem sap of pumpkin using multidimensional protein identification technology J Plant Physiol 2010 167 10 771 778 10.1016/j.jplph.2010.01.004 20138393

[B15] Schad M Lipton MS Giavalisco P Smith RD Kehr J Evaluation of two-dimensional electrophoresis and liquid chromatography tandem mass spectrometry for tissue-specific protein profiling of laser-microdissected plant samples Electrophoresis 2005 26 14 2729 2738 10.1002/elps.200410399 15971193

[B16] Koroleva OA Cramer R Single-cell proteomic analysis of glucosinolate-rich S-cells in Arabidopsis thaliana Methods 2011 54 4 413 423 10.1016/j.ymeth.2011.06.005 21708264

[B17] Vilaine F Palauqui JC Amselem J Kusiak C Lemoine R Dinant S Towards deciphering phloem: a transcriptome analysis of the phloem of *Apium graveolens* Plant J 2003 36 1 67 81 10.1046/j.1365-313X.2003.01855.x 12974812

[B18] Anstead JA Froelich DR Knoblauch M Thompson GA Arabidopsis P-protein filament formation requires both AtSEOR1 and AtSEOR2 Plant Cell Physiol 2012 53 6 1033 1042 10.1093/pcp/pcs046 22470058

[B19] Noll GA Ruping B Ernst AM Bucsenez M Twyman RM Fischer R Prufer D The promoters of forisome genes MtSEO2 and MtSEO3 direct gene expression to immature sieve elements in *Medicago truncatula* and *Nicotiana tabacum* Plant Mol Biol Reporter 2009 27 4 526 533 10.1007/s11105-009-0120-5

[B20] Pelissier HC Peters WS Collier R van Bel AJE Knoblauch M GFP tagging of sieve element occlusion (SEO) proteins results in green fluorescent Forisomes Plant Cell Physiol 2008 49 11 1699 1710 10.1093/pcp/pcn141 18784195 PMC2582178

[B21] Barratt DHP Derbyshire P Findlay K Pike M Wellner N Lunn J Feil R Simpson C Maule AJ Smith AM Normal growth of Arabidopsis requires cytosolic invertase but not sucrose synthase Proc Natl Acad Sci USA 2009 106 31 13124 13129 10.1073/pnas.0900689106 19470642 PMC2722301

[B22] Barratt DHP Kolling K Graf A Pike M Calder G Findlay K Zeeman SC Smith AM Callose synthase GSL7 is necessary for normal phloem transport and inflorescence growth in Arabidopsis Plant Physiol 2011 155 1 328 341 10.1104/pp.110.166330 21098675 PMC3075753

[B23] Sauer N Molecular physiology of higher plant sucrose transporters FEBS Lett 2007 581 12 2309 2317 10.1016/j.febslet.2007.03.048 17434165

[B24] Pommerrenig B Feussner K Zierer W Rabinovych V Klebl F Feussner I Sauer N Phloem-specific expression of yang cycle genes and identification of novel yang cycle enzymes in Plantago and Arabidopsis Plant Cell 2011 23 5 1904 1919 10.1105/tpc.110.079657 21540433 PMC3123959

[B25] Khan JA Wang Q Sjolund RD Schulz A Thompson GA An early nodulin-like protein accumulates in the sieve element plasma membrane of Arabidopsis Plant Physiol 2007 143 4 1576 1589 10.1104/pp.106.092296 17293437 PMC1851800

[B26] Keller A Nesvizhskii AI Kolker E Aebersold R Empirical statistical model to estimate the accuracy of peptide identifications made by MS/MS and database search Anal Chem 2002 74 20 5383 5392 10.1021/ac025747h 12403597

[B27] Zhang B Tolstikov V Turnbull C Hicks LM Fiehn O Divergent metabolome and proteome suggest functional independence of dual phloem transport systems in cucurbits Proc Natl Acad Sci USA 2010 107 30 13532 13537 10.1073/pnas.0910558107 20566864 PMC2922161

[B28] Aki T Shigyo M Yoneyama T Yanagisawa S Proteome analysis of rice phloem sap Plant Cell Physiol 2007 48 S125 S125

[B29] Kaundal R Saini R Zhao PX Combining machine learning and homology-based approaches to accurately predict subcellular localization in Arabidopsis Plant Physiol 2010 154 1 36 54 10.1104/pp.110.156851 20647376 PMC2938157

[B30] Ito J Batth TS Petzold CJ Redding-Johanson AM Mukhopadhyay A Verboom R Meyer EH Millar AH Heazlewood JL Analysis of the Arabidopsis cytosolic proteome highlights subcellular partitioning of central plant metabolism J Proteome Res 2011 10 4 1571 1582 10.1021/pr1009433 21166475

[B31] Moran PJ Thompson GA Molecular responses to aphid feeding in Arabidopsis in relation to plant defense pathways Plant Physiol 2001 125 2 1074 1085 10.1104/pp.125.2.1074 11161062 PMC64906

[B32] Anstead J Samuel P Song N Wu C Thompson GA Goggin F Activation of ethylene-related genes in response to aphid feeding on resistant and susceptible melon and tomato plants Entomologia Experimentalis Et Applicata 2010 134 2 170 181 10.1111/j.1570-7458.2009.00945.x

[B33] Guerrieri E Digilio MC Aphid-plant interactions: a review J Plant Interact 2008 3 4 223 232 10.1080/17429140802567173

[B34] Dinant S Bonnemain J-L Girousse C Kehr J Phloem sap intricacy and interplay with aphid feeding C R Biol 2010 333 6–7 504 515 20541162 10.1016/j.crvi.2010.03.008

[B35] Moran PJ Cheng YF Cassell JL Thompson GA Gene expression profiling of Arabidopsis thaliana in compatible plant-aphid interactions Arch Insect Biochem Physiol 2002 51 4 182 203 10.1002/arch.10064 12432519

[B36] Barth C Jander G Arabidopsis myrosinases TGG1 and TGG2 have redundant function in glucosinolate breakdown and insect defense Plant J 2006 46 4 549 562 10.1111/j.1365-313X.2006.02716.x 16640593

[B37] Mahajan SK Chisholm ST Whitham SA Carrington JC Identification and characterization of a locus (RTM1) that restricts long-distance movement of tobacco etch virus in *Arabidopsis thaliana* Plant J 1998 14 2 177 186 10.1046/j.1365-313X.1998.00105.x 9628015

[B38] Chisholm ST Parra MA Anderberg RJ Carrington JC Arabidopsis RTM1 and RTM2 genes function in phloem to restrict long-distance movement of tobacco etch virus Plant Physiol 2001 127 4 1667 1675 10.1104/pp.010479 11743111 PMC133571

[B39] Doxey AC Yaish MWF Moffatt BA Griffith M McConkey BJ Functional divergence in the Arabidopsis beta-1,3-glucanase gene family inferred by phylogenetic reconstruction of expression states Mol Biol Evol 2007 24 4 1045 1055 10.1093/molbev/msm024 17272678

[B40] Ahmed SU Rojo E Kovaleva V Venkataraman S Dombrowski JE Matsuoka K Raikhel NV The plant vacuolar sorting receptor AtELP is involved in transport of NH2-terminal propeptide-containing vacuolar proteins in Arabidopsis thaliana J Cell Biol 2000 149 7 1335 1344 10.1083/jcb.149.7.1335 10871276 PMC2175142

[B41] Grbic V Bleecker AB Ethylene regulates the timing of leaf senescence in Arabidopsis Plant J 1995 8 4 595 602 10.1046/j.1365-313X.1995.8040595.x

[B42] Dal Santo S Stampfl H Krasensky J Kempa S Gibon Y Petutschnig E Rozhon W Heuck A Clausen T Jonak C Stress-induced GSK3 regulates the redox stress response by phosphorylating glucose-6-phosphate dehydrogenase in Arabidopsis Plant Cell 2012 24 8 3380 3392 10.1105/tpc.112.101279 22885737 PMC3462638

[B43] Jiang Y Yang B Harris NS Deyholos MK Comparative proteomic analysis of NaCl stress-responsive proteins in Arabidopsis roots J Exp Bot 2007 58 13 3591 3607 10.1093/jxb/erm207 17916636

[B44] Xin ZY Zhao YH Zheng ZL Transcriptome analysis reveals specific modulation of abscisic acid signaling by ROP10 small GTPase in Arabidopsis Plant Physiol 2005 139 3 1350 1365 10.1104/pp.105.068064 16258012 PMC1283771

[B45] Kiyosue T Yamaguchishinozaki K Shinozaki K Characterization of 2 cDNAs (ERD10 and ERD14) corresponding to genes that respond rapidly to dehydration stress in *Arabidopsis Thaliana* Plant Cell Physiol 1994 35 2 225 231 8069491

[B46] Kim SY Nam KH Physiological roles of ERD10 in abiotic stresses and seed germination of Arabidopsis Plant Cell Rep 2010 29 2 203 209 10.1007/s00299-009-0813-0 20054552

[B47] Marondedze C Turek I Parrott B Thomas L Jankovic B Lilley KS Gehring C Structural and functional characteristics of cGMP-dependent methionine oxidation in Arabidopsis thaliana proteins Cell Commun Signal 2013 11 1 10.1186/1478-811X-11-1 23289948 PMC3544604

[B48] Mustafiz A Singh AK Pareek A Sopory SK Singla-Pareek SL Genome-wide analysis of rice and Arabidopsis identifies two glyoxalase genes that are highly expressed in abiotic stresses Funct Integr Genomics 2011 11 2 293 305 10.1007/s10142-010-0203-2 21213008

[B49] Xiong LM Lee HJ Ishitani M Zhu JK Regulation of osmotic stress-responsive gene expression by the LOS6/ABA1 locus in Arabidopsis J Biol Chem 2002 277 10 8588 8596 10.1074/jbc.M109275200 11779861

[B50] Cai M Qiu DY Yuan T Ding XH Li HJ Duan L Xu CG Li XH Wang SP Identification of novel pathogen-responsive cis-elements and their binding proteins in the promoter of OsWRKY13, a gene regulating rice disease resistance Plant Cell Environ 2008 31 1 86 96 17986178 10.1111/j.1365-3040.2007.01739.x

[B51] Santagata S Boggon TJ Baird CL Gomez CA Zhao J Shan WS Myszka DG Shapiro L G-protein signaling through tubby proteins Science 2001 292 5524 2041 2050 10.1126/science.1061233 11375483

[B52] Reitz MU Bissue JK Zocher K Attard A Huckelhoven R Becker K Imani J Eichmann R Schafer P The subcellular localization of tubby-like proteins and participation in stress signaling and root colonization by the Mutualist *Piriformospora indica* Plant Physiol 2012 160 1 349 364 10.1104/pp.112.201319 22751378 PMC3498949

[B53] Schobert C Baker L Szederkenyi J Grossmann P Komor E Hayashi H Chino M Lucas WJ Identification of immunologically related proteins in sieve-tube exudate collected from monocotyledonous and dicotyledonous plants Planta 1998 206 2 245 252 10.1007/s004250050396

[B54] Hafke JB Ehlers K Foller J Holl SR Becker S van Bel AJ Involvement of the sieve element cytoskeleton in electrical responses to cold shocks Plant Physiol 2013 162 2 707 719 10.1104/pp.113.216218 23624858 PMC3668064

[B55] Cosgrove DJ Growth of the plant cell wall Nat Rev Mol Cell Biol 2005 6 11 850 861 10.1038/nrm1746 16261190

[B56] Cosgrove DJ Li LC Cho HT Hoffmann-Benning S Moore RC Blecker D The growing world of expansins Plant Cell Physiol 2002 43 12 1436 1444 10.1093/pcp/pcf180 12514240

[B57] Zhao CS Craig JC Petzold HE Dickerman AW Beers EP The xylem and phloem transcriptomes from secondary tissues of the Arabidopsis root-hypocotyl Plant Physiol 2005 138 2 803 818 10.1104/pp.105.060202 15923329 PMC1150398

[B58] Bordenave M Breton C Goldberg R Huet JC Perez S Pernollet JC Pectinmethylesterase isoforms from *Vigna radiata* hypocotyl cell walls: kinetic properties and molecular cloning of a cDNA encoding the most alkaline isoform Plant Mol Biol 1996 31 5 1039 1049 10.1007/BF00040722 8843946

[B59] Harriman RW Tieman DM Handa AK Molecular cloning of tomato pectin methylesterase gene and its expression in Rutgers, ripening inhibitor, nonripening and never ripe tomato fruits Plant Physiol 1991 97 1 80 87 10.1104/pp.97.1.80 16668419 PMC1080966

[B60] Guenin S Mareck A Rayon C Lamour R Ndong YA Domon JM Senechal F Fournet F Jamet E Canut H Identification of pectin methylesterase 3 as a basic pectin methylesterase isoform involved in adventitious rooting in *Arabidopsis thaliana* New Phytologist 2010 192 1 114 126 21692803 10.1111/j.1469-8137.2011.03797.x

[B61] Raiola A Lionetti V Elmaghraby I Immerzeel P Mellerowicz EJ Salvi G Cervone F Bellincampi D Pectin methylesterase is induced in Arabidopsis upon infection and is necessary for a successful colonization by necrotrophic pathogens Mol Plant Microbe Interact 2011 24 4 432 440 10.1094/MPMI-07-10-0157 21171891

[B62] Walz C Juenger M Schad M Kehr J Evidence for the presence and activity of a complete antioxidant defence system in mature sieve tubes Plant J 2002 31 2 189 197 10.1046/j.1365-313X.2002.01348.x 12121448

[B63] MacMillan CP Mansfield SD Stachurski ZH Evans R Southerton SG Fasciclin-like arabinogalactan proteins: specialization for stem biomechanics and cell wall architecture in Arabidopsis and Eucalyptus Plant J 2010 62 4 689 703 10.1111/j.1365-313X.2010.04181.x 20202165

[B64] Liu C Li LC Chen WQ Chen X Xu ZH Bai SN HDA18 Affects cell fate in Arabidopsis root epidermis via Histone acetylation at four kinase genes Plant Cell 2013 25 1 257 269 10.1105/tpc.112.107045 23362208 PMC3584540

[B65] Bourgis F Roje S Nuccio ML Fisher DB Tarczynski MC Li CJ Herschbach C Rennenberg H Pimenta MJ Shen TL S-methylmethionine plays a major role in phloem sulfur transport and is synthesized by a novel type of methyltransferase Plant Cell 1999 11 8 1485 1497 10449582 10.1105/tpc.11.8.1485 PMC144290

[B66] Tan Q Zhang L Grant J Cooper P Tegeder M Increased phloem transport of S-methylmethionine positively affects sulfur and nitrogen metabolism and seed development in Pea plants Plant Physiol 2010 154 4 1886 1896 10.1104/pp.110.166389 20923886 PMC2996030

[B67] Pallas V Gomez G Phloem RNA-binding proteins as potential components of the long-distance RNA transport system Frontiers Plant Sci 2013 4 130 10.3389/fpls.2013.00130 PMC3650515 23675378

[B68] Peterman TK Ohol YM McReynolds LJ Luna EJ Patellin1, a novel Sec14-like protein, localizes to the cell plate and binds phosphoinositides Plant Physiol 2004 136 2 3080 3094 10.1104/pp.104.045369 15466235 PMC523369

[B69] Barnes A Bale J Constantinidou C Ashton P Jones A Pritchard J Determining protein identity from sieve element sap in Ricinus communis L. by quadrupole time of flight (Q-TOF) mass spectrometry J Exp Bot 2004 55 402 1473 1481 10.1093/jxb/erh161 15181102

[B70] Aoki K Fujimaki S Fujiwara T Hayashi H Yamaya T Sakakibara H Detection of systemically moved phloem proteins which introduced into rice leaf by using insect-stylet method Plant Cell Physiol 2002 43 S192 S192

[B71] Fukuda A Okada Y Suzui N Fujiwara T Yoneyama T Hayashi H Cloning and characterization of the gene for a phloem-specific glutathione S-transferase from rice leaves Physiol Plant 2004 120 4 595 602 10.1111/j.0031-9317.2004.0253.x 15032821

[B72] Froelich DR Mullendore DL Jensen KH Ross-Elliott TJ Anstead JA Thompson GA Pelissier HC Knoblauch M Phloem ultrastructure and pressure flow: sieve-element-occlusion-related agglomerations do not affect translocation Plant Cell 2011 23 12 4428 4445 10.1105/tpc.111.093179 22198148 PMC3269875

[B73] Martin T Frommer WB Salanoubat M Willmitzer L Expression of an Arabidopsis sucrose synthase gene indicates a role in metabolization of sucrose during phloem loading and ion sink organs Plant J 1993 4 2 367 377 10.1046/j.1365-313X.1993.04020367.x 8220487

