# Supplementary material for: The broccoli (Brassica oleracea) phloem tissue proteome
Source: BMC Genomics. 2013 Nov 7;14:764. doi: 10.1186/1471-2164-14-764 (PMC3833381; doi:10.1186/1471-2164-14-764)
Supplement: Additional file 1: Table S1 — Primer sequences used to amplify gene fragments from Arabidopsis genes identified from phloem protein sequences. [file 1471-2164-14-764-S1.docx]

**Table s1: Primer sequences used to amplify gene fragments from Arabidopsis Genes identified from phloem protein sequences.**

| Gene codes (from The Arabidopsis Information Resource TAIR) | Primer sequence - Forward | Primer sequence - Reverse |
| --- | --- | --- |
| AT1G04410 | TCGTTACTGGAGCTGCAGGACAAAT | CGGCATCAGTTGTAGCAACAACACC |
| AT1G04750 | ACAACTGCGACGGTCATACC | CATCAGGATGATCCATGCAA |
| AT1G06680 | AAGTATCTCCTGCTGATGCC | TTCTTGTCGGTAGGAGTGAC |
| AT1G07930 | CCCTTGGTGTCAAGCAGATG | GAGTTGGTCCCTTGTACCAG |
| AT1G11840 | GCTCACATATAACTATGGTG | AGCTCAAAAGTGTAGCCATC |
| AT1G20440 | CCGATCCAACAGCTCTTCTTCCTCT | ATGCTCCACCACACTCTCCGACA |
| AT1G20450 | CCGATCCAACAGCTCTTCTTCCTCT | TGGTGCTGGCATGGTGGTGA |
| AT1G20620 | GAGAAAGAGGTCCGGTTCTTCTTGAGGATT | CAGCACAAGTGAGGTTTGAAATGTCATG |
| AT1G22530 | CCATCCAAATCCGCTGAGACCA | CCGTAGCTCACGTCCGCACCCAAAA |
| AT1G23290 | TATCGGTAAGCACCGTAAGCATCCC | TTATCCTTGCTCGATTTCGCCTTCA |
| AT1G26770 | ATGGGTGGTGCTTGTGGATATGGT | GCTAACGCGTTATTTGGCGGGC |
| AT1G32060 | ACCCTCCGGAGCTTATTCAGCCT | TCCGCGTACTGCTTTTGCGG |
| AT1G32470 | TTCTCCTCAGTGTTGGAAGG | CTCACTTGTGGCCTTAACAC |
| AT1G35720 | TAACGATTTCGAGAGAGCTATCTTGTTGTG | AGCGTGAAGCAGCTGCGTTGAT |
| AT1G41830 | ACACGGATTTGAAGTCTCGTCTTGATAGA | CGGTATGTTTTTCCTTGTTCAACGTTTAAG |
| AT1G48850 | AGTCTCTGAAGGAATGACGA | GAGCAACTCTTCCAATGGTC |
| AT1G51760 | TGCACTTGCTATGCAGGAAATGGTG | TCTTCTTTGCACCTCCTCCTCCTTC |
| AT1G52400 | GCAGCATTTCAGGTTGAAGGAGCTG | TTCTGGGCCACGCAATAGAAAGTC |
| AT1G53070 | TCTTAGCTATCTTCACCTCTCAAGTCACCA | AGGAATGTTGTCTCGAGTCAATGCG |
| AT1G53240 | ACACTCCTGGAGTTGCTGCTGATGT | TGATCATATTAATAAGCGCATGTGG |
| AT1G54410 | ATGGCAGGACTCATCAACAAGATCG | TGTCCTTCTTCTTTTTCTTCTCACCGTC |
| AT1G56070 | TATTGCTCATGTCGACCACG | CTCTGGCTCCAGTGAAACTC |
| AT1G56340 | ACACAGCTGGAAATTGGTCT | GCTGTAAGGAGTGTCTCCTC |
| AT1G65930 | CGAGATGGATGGTGATGAAATGACC | CATCTGGAGTGATGGTGGCACACTT |
| AT1G65960 | AATGTTCAAGTTTGCTGGGAGAAATTCG | CCCAACCAATACCAGCATAGACTAGTCCAT |
| AT1G67280 | CTCTCGCACTCTCCATGTTCCTCAA | TCCGGTCCATATCGCCAACACG |
| AT1G70490 | TCGGAGAGATTGTCACCACC | TCCTCATTCAGCATCCTGTG |
| AT1G72150 | CTAAAGAGCTATTCATCAATGTCCCATGG | TCGGCAGATTTGGATGGACCAG |
| AT1G72160 | TTGTCTTCAAACAGGCTTTTATCAATGTTC | AATAGGGTTTCAGCTGAACGCGAAG |
| AT1G73370 | ACATTCTCACTTGTACTCAGGAAGCAGCA | CCTTGAGCTTTAAGTAATCAGTGGCTGTG |
| AT1G75780 | CCGCACTCTCAAACTCAGCACTCC | AGCTCCGGGACTGTGAGAGAGATGT |
| AT1G76030 | GCGAGAAAGCAGTTGTGCAGGTTT | CACTGGGGTTGATTGAACTTCCTGA |
| AT1G76180 | AAGCTTCACCGAAGCGACAGTTCTT | ATGCGCTTCTTCCACAGGAGGAG |
| AT1G77120 | CGATGCAAAGCTGCTGTGGCA | CAACAATCCCTCCAGCTTCATGGC |
| AT1G78900 | GCCACCATCCAAGTTTACGAGGAA | GGGCTGGAACTCCCAAAGACAATC |
| AT1G79340 | TGATTTTAGAGATCTTGTGGACAAGGTTCC | AACTTCCTAAACCCGAATCTTGAAGAAGAT |
| AT1G79550 | TTCTCTGCAGTCACTTGGGCCG | CTCTTCCTCGGCGTAGAACCTCACA |
| AT1G80660 | GCCTCGAGATCTTCGGTCCTAACAA | CGGCGTTGTTTTCTTCAATGAAGC |
| AT2G01140 | TAAAACCGCCAAAAGCATTGCATC | CGCAAGCAATCGACAAAGGTCTTAC |
| AT2G05920 | TGTCAGGTACGTCAATGTCATGCCC | ACATGGCCCGAGCCGTGAGCATAT |
| AT2G05990 | TGGGACTTGGGTTCCTGCACTTAA | CTTCCTGTACGGTCCAGTTTGACGA |
| AT2G17130 | TGCCTGGTTTAGCTTCTCGTCATGA | TGCTCTGGTCTAGCCACAAGTTGCA |
| AT2G18020 | GCTCACAACCCTGACAGCGACACT | CCATGAGGATGCTCCACTGGATTC |
| AT2G19760 | GCTGTGATCCGAGGGAAGAAGGG | GCCTTTCGACAACCAAGTTGCATTG |
| AT2G20140 | CGTTGGCATTTTGTCTTTCGTTGAT | TGCCTGGCTCACCATACAAGATCA |
| AT2G21170 | CATGGCTGGATCCGGAAAGTTTT | CCCAACCCAAGAGTTCTGACCTGA |
| AT2G21330 | AGTCGACAAGGGTTTGGTGCCA | CGAGCACCTTGTTGGTAGTAAGCAGC |
| AT2G21430 | CGTGAAAAACCAGGGATCTTGTGG | CCCTCCGGTTTTAAGCGTGTATTCA |
| AT2G21660 | TGAGACTGCCTTCGCTCAATACGG | TCCACCGGAGTAACCTCCACCA |
| AT2G22170 | CGCATTAGCCGACGATGAAGCA | GAAGACATGGTGCTTTCCCACTGAA |
| AT2G24200 | TTAGCTTCAGGCATAGTACT | TACAGCAGGAGTAAGCACAT |
| AT2G25970 | TGGCGAGAACCGTATGAGAAACTCA | GATGGTGGCACAGAGGACTGATCAT |
| AT2G26100 | TCTAAGGTTGGAACAAGCGACTGGA | CGTCGCAAGTCGATCTGGTCTCA |
| AT2G27860 | GAGTGGTCGGATCCAGTTTCATCG | GCAGGATCATCACGCAGAGGATG |
| AT2G28950 | GGAACAATGGGAGGAGCTTGTGG | CGGAGGACAGAAATTAGTCGCAGTG |
| AT2G30860 | GCCTGCTTATCTCGCTCTACAGCCT | CGTTAAGTTCAATAGCGGTGGGTGG |
| AT2G36530 | TGAGCTGAGGGATGGAGGATCTGA | ACAGCCCCAGCTTTGCAGACAG |
| AT2G37270 | TGCCATTGTTAACAGTGGTCCACG | CCCTTTGCAGCATTGATGAGTTCAT |
| AT2G39460 | AATCATTTCAAGCCATGTCTCCGG | CAACTTGTTCCTTGGAGTAGCGCTG |
| AT2G41530 | CCCTCCTTCTGCTTCTTCTTCCCA | CGCCAGTTCTTCCACTTTTCCTGA |
| AT2G41740 | CTGGCCAGAAACCAGGGACTGAAAT | GGCCTCCAAGAACTGCATCGAGT |
| AT2G45470 | CTTCCACCGTGAGTAGCCACAACAT | GGACGAGGAGGCTTAAAGCGCTT |
| AT2G47900 | TCCTATCTCCCTCAAACAGCCGG | TCCTCGAGAGACATCATCGCAGTTT |
| AT3G01280 | GAGATGTCGCTTTTCAGTCGAGGC | AAGACATTGGAGCCAATCACACCAG |
| AT3G01670 | ACATCTTCAAGTCTCATGTCCCATC | GCAGTCGATCTCGCAAGAGATT |
| AT3G01680 | ACCATCTCGCTGAGACCTTGAGG | GGCCGTGAGAATCTTCATGTTATCA |
| AT3G02090 | GAAATGCAAGAGGTGGAGGGACAA | AACAGCTCCTGCAGCAGCAATCA |
| AT3G02230 | GCTTCGGCTACATGGTCTCCAAGA | AGGGATGTTGAGCCACAGACCGT |
| AT3G03780 | ACACCAACTACCATTACATCGTCCCAG | GAGAAGATCAAATGACTTGTCAACACCCTT |
| AT3G07930 | AACATCTGGTGCACAGACGCGG | TGCAGTTGAGTGACATGAGTCCAGC |
| AT3G08030 | ACCAGAGGGAACTTGGTGAAGAATGGA | GCATGTCCAAAGGGGACATTAAAGTACTTA |
| AT3G09840 | CCCCAGCTGAATCTTCAGACTCGA | GCAAGAGCAATGCAGACAGTGTCCT |
| AT3G10740 | CGCTATTTGGGATTTTCTTCGAGGA | CCGATGGACAACCTTTGCTATCACA |
| AT3G11130 | CAGATGCCTGAGCAGGTTGCTTTT | GGCAATTCCAATCAAGACCAACCA |
| AT3G12020 | ACCGAGTATCTCTCATTTGT | CCTGTTTAAGCTGTTCCAAC |
| AT3G12390 | TGACGAGGCTGATGGACTAGATGGA | CCTCGATCTTCGCCTCTCCAAAG |
| AT3G14310 | TTGAAGGGGCAGATACACGTAGAGCA | CTACAGCCACCGTAGTTTCCTGATTCTC |
| AT3G14990 | CCGCAACTATGGCTTCATTTACGAA | GGGGAGCACAATAAGGTCGAAAACA |
| AT3G16640 | TGGTGTACCAAGATCTTCTCACCGG | TGCTCCTGAAGTCTGAAGGTGTCGA |
| AT3G17240 | GGCTGTATTCCTTCAAAGGCCCTTC | TGGGGACAGAAACTTACCATAGCCC |
| AT3G17820 | CCACCGGGAAAATCATCGCC | TCACCAGGATGTTGTTGCCTTTCC |
| AT3G18780 | TTCCGCTCTTTCTTTCCAAGCTCA | CCCAGTTGCTAACAACACCATGCTC |
| AT3G20820 | AGCTCACCGAACCGGTTACATGAC | CGGAGATTCGGTTATCGGCTACG |
| AT3G21100 | CAGCGTAATGCGCATAGATCAGGA | TGCACTGGTCCAAAATTGCCAAA |
| AT3G21770 | TGCTTCGTTAGGGGATGTGACGG | TGCGCAAGAAACAGTCTTAGGACACAC |
| AT3G25230 | CTGGCGTACCGTTCGTGGGATGAT | CCCTGAGCTTGGTGAGTTGCTTCTT |
| AT3G26340 | CAATTCTGGCACAGAAATCTGGGAA | CGTATGGTGAACCTGAACCGACAGA |
| AT3G29360 | TTGAGAAACATGTTAGGGAAGCTGATATTG | CAACGACAATCTTATCCGATACCGAAA |
| AT3G32980 | GCGCAGATATACTTACCATCGCAGC | GGGTCGGGTAAACCAGTGTTGCTAA |
| AT3G48930 | CCTCGTGAAGCCATTGATGGAGC | ATGGTGAGACATGAGCCGGAATGT |
| AT3G50820 | AGCCACCTCTGCTCTCGTTGTCTCG | ACGGTGAAGGAAGTGGGCTCGA |
| AT3G51880 | CCAGTTGATGACAGAAAGGTGGGAA | AGCTTTCCCAACAGCAGAGACGG |
| AT3G52880 | CCATTGCTGTGTTGGTAGTGGTGG | GCTTCAACCAATTTGTCGGCATCA |
| AT3G55440 | CTGGTGAAGTGAGTGCGGAGATGC | CATCCATGGTCGATCCAGCTTCC |
| AT3G57020 | TGCCTACACATCTCCTCACAGAGGG | CAGCCACGAAAAATACGTCCCTGA |
| AT3G61260 | CAAATTTTCGATGACTCCAAAGCCC | GCTGCTTTCTTGCTGTTTTCCCAA |
| AT3G61430 | TGGTATCTCCGGTGGACACATCAAC | CCTTGGTGTAACCATGAGCCACAGT |
| AT4G01690 | TGGTGGTAGATAGTGGTTTG | AGATTCTTCACGACCTGGAG |
| AT4G01850 | CGCCGCAGCTTTAAGAAATGGA | TCGTAGTCAATCGTAGCCTTGGTGG |
| AT4G02450 | CGTCACCGTTCGTGTTTCCTCTTC | CCGGCTCTGCTTTCTCAATTATGC |
| AT4G09320 | CGGTGAAGTCATCTGCAGGTTTGAG | CTCAGAAGCTGCTGGGTTGGTAGC |
| AT4G11010 | GCTTCTTCTCTGAAGGACGAGCCAT | GTTCGTTCCATCTCAGCAGCAAGAA |
| AT4G11150 | GCGGAAGAGAAAGCAAACGAGATCT | CTTAGCTGCTTGGTCTTTCATGGCA |
| AT4G11290 | CTTCGTTCGGGGTTGTGATGGTT | CATGTCGGTCCACCAATAGCAACA |
| AT4G12730 | TTTCTCCACTTTCAACCACTATCTCTCAGC | GGAGAGAGAGAATGTTGCGGATTTGG |
| AT4G12880 | GAGACCAACACAACATTCTTGAGGTAAACA | AACGCAACCACCTCTTCCATCAAGTA |
| AT4G13340 | CTACAAGCTTGGAAACAAGCGATTCTCTCT | CGTGATTGAGATCGATTCCGGC |
| AT4G13940 | GCTTTACCAGATGCAGCAAAATGGA | CTTGAAGGGCACAGATGGGATCAA |
| AT4G14880 | TCATGGCCTCGAGAATTGCTAAAGA | CCACTTGTTGGCTCAATCAGCACA |
| AT4G14960 | GGAAATGCTTGCTGGGAGCTTTACT | GGCGAAATTGTTAGCAGCGTCTTC |
| AT4G17260 | CGCAGATCATCTCGACGTTAATGCT | CAATGGCCCAAGAAGTGTAACCCTT |
| AT4G18030 | CATCGACCAGGAGATTAGAA | AGGTTTGGGATCAGCTTTAT |
| AT4G19410 | TCTCTAACCAAACAAGAAGAGGACTCCATT | TGCAATCTTCTTGAAATCCTAAATGGCTAA |
| AT4G20440 | GGCGATGCCTTGTTCTTCTTCTGA | ACTCAGGATGAGCTTCGATGAAGCA |
| AT4G21770 | TCCAATTCTGGATACCATGA | TTTAGGACATACAAGAGCGT |
| AT4G23590 | CAAGGAAAGCCTATTTTGCCTCCAC | GCTCGATTGCTTGTTTGCATCCA |
| AT4G24190 | CCGTTTTGTTCCTTTTCTCGCTTCT | CCGAGAAACCTCAGCTTGGAACTCA |
| AT4G25630 | CCTCTAACTGGAAGTGGTGGTGGGT | TCCTCCTTTCATGCCTCCACGG |
| AT4G26270 | CGCATCTCTCCGATTACCTTCCTGG | GCCAGGACAAAGACCTCCACAGGT |
| AT4G29700 | TCATCGTAGCCACCATCGCAGC | GGGATTAAACCGTGTTTGGCCTCA |
| AT4G31300 | GCCATTGGAGGCTCTGGTTCGA | TGGCTCCATCACGGGCGAT |
| AT4G33680 | TGGCATAGGGGATGATGACGTTTT | TTATATCTGTCCTGCCAACGGTGGA |
| AT4G34490 | AAGCAAACTCAGAAGCCTGACCTCG | TTGCCAACTTTCTTCCACATGAGCT |
| AT5G07030 | GTGTAAGCAGGTACCAAACCCCACG | AAGCCCAACAAGCCTTGAGGTGG |
| AT5G07440 | GGGTTTGGATTCGAAGATCGAGAGA | TCCACCTTTAGCACCACCATATGGA |
| AT5G09650 | TCCTCGATGGTTCTGGAAAGAAGGT | CCCATGTTTGAGGAAGCAACCCA |
| AT5G09810 | TCTTCTTCAAGTGAAAAATGGC | ACTGAGCTTCATCACCAACG |
| AT5G11420 | CCTCCTCGGCCCACCAATAAGA | TTTGCCTGCCACTAGCTCCACG |
| AT5G11770 | GGCTCTTCGCAAGGTTTATGACCA | GCGCCTGTTGATTTTCTTCTGAAGC |
| AT5G15650 | ATTGCTTCGTTGCCAAGGATCCA | TTGAGCCAAAGACCATGGGAAACA |
| AT5G16450 | CCGTCAATTCATCGAGGAGAAAGGA | TTTTATCGGATGAGAGGCCAAAGCT |
| AT5G16590 | CTGTTACACGGGAACAAAGGAAGTGG | AGAGACCTTAGCTTCAAAGGATTCTGACAA |
| AT5G17310 | CCCAAACCCGAAGGAAGTTGACG | TGGGTTTGTGGGGTTTGTTCTAGCT |
| AT5G19440 | CCGTGATCCCAGTGATCCGAAA | ACCGCAGGATCAATAAGTTCAGCCT |
| AT5G19510 | TCGCCGGAAAAACCTACATCTCC | TGCAGGTGCCTCAGCTTCAACA |
| AT5G19760 | TGGATCATTCAAGTTGCTGACTGCA | GGTAAGCGCATGGAAAGCATTGG |
| AT5G20080 | TTATCCTTGTATGCGGTCCT | GATCCGTTTTGCTTTGTTTA |
| AT5G20830 | TGCCTTGCTTTCCAGGGTTGAA | AAGTATTCCCAAACACCAGGCCTTG |
| AT5G20870 | CCGACATAAGATATGTAGCGGTGGGG | CGTAGACGTCGGCATTAAGAGGAACC |
| AT5G25980 | TTACCAGATCGAAGGCGGCAGA | GTCCGGTATGAGTCACAAGTAGTGTCTCC |
| AT5G26710 | TCTCCGACGGGAGGAAATTGAATG | GCCAACAAGAAACGTGCTACTCTCG |
| AT5G37510 | TGCCTGCGCTTCCTGGGATGAA | CCCAGCAACTTCAGAAGCAAATCG |
| AT5G38430 | CTGCATGAAGGTGTGGCCACCA | CACATTGTCCAGTACCGTCCATCGT |
| AT5G39570 | TATGGCCGCAAGAAATATGGTGGC | CAAACACAACACCAACGTCGGAAG |
| AT5G40760 | TCTTGGTGCTTCTGGTGATCTTGCT | CTCCTCAGCATCATAAGGGCCACTC |
| AT5G43830 | TCGTGTTCCTAGATTATTTG | TTCCATGGAAATCTCTAACA |
| AT5G44340 | CAGCGATTGTCTTCAAGGTTTCCAA | CGTTTTCGACAAGCTGATGCACAG |
| AT5G45280 | GGCTCAGGAGTGAACAATTGGATCG | TGAAAGATGAACCATCGCAATACCG |
| AT5G46290 | GCTCTTGAAAGTGCCAATCTTGGTG | GCAGCAGCGTAAAAGCAGTAATTCG |
| AT5G49360 | TCAATGGGTACATTGTCTCAGATTGTGAC | GCACCTTCCGTGAAAATCGCCA |
| AT5G49460 | CGACTCGGGTCGTGACCAGTTTT | GCGATTTCCTCCTGCCCGAA |
| AT5G60360 | TGCTGATTTGACATGGCAAGAGTTTC | CCCTGATCTTTGACCGGACTAACGATA |
| AT5G61790 | TCGTTTCGTTCCAGAAGCTTTGCT | TCCTTCCTTGAGGTTTAGAGGCTCG |
| AT5G62690 | TGACTGTCTCCAAGGGTTCCAGGTT | CCAAAGCTGGGGGTAGTGAGTTTGA |
| AT5G64740 | CGATGAGAATGCCCGAATAAGATCA | GACGTTTGAAACGGGTTTTGCACT |
| AT5G65760 | GCCTTGCTTGTTTTCCCCGAGC | GCCTCATCCATGCTGCTAACATTCC |
| AT5G66570 | TTGCCACCTCTGCTCTCGTCGT | CGGTGAAGGAAGTAGGCTCGAAGC |
| AT5G66760 | TTTGATGGCGTCTCTGGATCTCG | TGCAGCATTAATACCACCCTGAGCA |
