# Supplementary material for: The broccoli (Brassica oleracea) phloem tissue proteome
Source: BMC Genomics. 2013 Nov 7;14:764. doi: 10.1186/1471-2164-14-764 (PMC3833381; doi:10.1186/1471-2164-14-764)
Supplement: Additional file 4: Table S4 — Functional classification of phloem proteins compared to the Arabidopsis whole genome using GO slim annotations, analyzed by the proportion of annotation counts and separated into GO molecular functions and Go biological process expressed in number of proteins and percentages of the total number of proteins identified. [file 1471-2164-14-764-S4.doc]

| **GO molecular functions** | **Phloem** | | **Whole genome** | |
| --- | --- | --- | --- | --- |
|  | **Number of proteins** | **% of total** | **Number of proteins** | **% of total** |
| other enzyme activity | 223 | 21.0 | 5715 | 11.5 |
| other binding | 189 | 17.8 | 6488 | 13.0 |
| hydrolase activity | 136 | 12.8 | 4921 | 9.9 |
| nucleotide binding | 113 | 10.7 | 3980 | 8.0 |
| protein binding | 92 | 8.7 | 6439 | 12.9 |
| transferase activity | 76 | 7.2 | 6683 | 13.4 |
| structural molecule activity | 61 | 5.8 | 560 | 1.1 |
| DNA or RNA binding | 47 | 4.4 | 3909 | 7.9 |
| transporter activity | 45 | 4.2 | 2551 | 5.1 |
| other molecular functions | 27 | 2.5 | 1308 | 2.6 |
| unknown molecular functions | 20 | 1.9 | 11072 | 22.2 |
| kinase activity | 19 | 1.8 | 3721 | 7.5 |
| nucleic acid binding | 9 | 0.8 | 1610 | 3.2 |
| transcription factor activity | 3 | 0.3 | 1680 | 3.4 |
| receptor binding or activity | 0 | 0 | 227 | 0.5 |
| **GO biological Process** | **Phloem** | | **Whole genome** | |
|  | **Number of proteins** | **% of total** | **Number of proteins** | **% of total** |
| other cellular processes | 530 | 27.1 | 17991.0 | 24.3 |
| other metabolic processes | 474 | 24.2 | 15763.0 | 21.3 |
| response to stress | 198 | 10.1 | 3836.0 | 5.2 |
| response to abiotic or biotic stimulus | 183 | 9.3 | 3488.0 | 4.7 |
| protein metabolism | 132 | 6.7 | 4935.0 | 6.7 |
| other biological processes | 124 | 6.3 | 2895.0 | 3.9 |
| cell organization and biogenesis | 80 | 4.1 | 1904.0 | 2.6 |
| transport | 75 | 3.8 | 3485.0 | 4.7 |
| developmental processes | 72 | 3.7 | 3763.0 | 5.1 |
| electron transport or energy pathways | 38 | 1.9 | 389.0 | 0.5 |
| unknown biological processes | 21 | 1.1 | 10921.0 | 14.7 |
| signal transduction | 15 | 0.8 | 1652.0 | 2.2 |
| DNA or RNA metabolism | 11 | 0.6 | 772.0 | 1.0 |
| transcription,DNA-dependent | 5 | 0.3 | 2249 | 3.0 |
