# Supplementary material for: The broccoli (Brassica oleracea) phloem tissue proteome
Source: BMC Genomics. 2013 Nov 7;14:764. doi: 10.1186/1471-2164-14-764 (PMC3833381; doi:10.1186/1471-2164-14-764)
Supplement: Additional file 5: Table S5 — Functional classification of phloem proteins by extraction process (soluble fraction, Chapso fraction or SDS fraction) analyzed by the proportion of annotation counts and separated into GO cellular components, GO molecular functions and Go biological process expressed in number of proteins and percentages of the total number of proteins identified. [file 1471-2164-14-764-S5.doc]

| **GO Cellular Component** | **Soluble** | | **SDS** | | **CHAPSO** | |
| --- | --- | --- | --- | --- | --- | --- |
|  | **Number of proteins** | **% of total** | **Number of proteins** | **% of total** | **Number of proteins** | **% of total** |
| other intracellular components | 265 | 23.0 | 228 | 23.9 | 136 | 26.1 |
| other cytoplasmic components | 198 | 17.2 | 162 | 17.0 | 99 | 19.0 |
| [cytosol](http://www.arabidopsis.org/servlets/Search?type=gene&action=search&taxon=1&origin=locigoslim&origin_identifier=2944818&slim_name=cell wall) | 137 | 11.9 | 57 | 6.0 | 33 | 6.3 |
| [chloroplast](http://www.arabidopsis.org/servlets/Search?type=gene&action=search&taxon=1&origin=locigoslim&origin_identifier=2944818&slim_name=chloroplast) | 86 | 7.5 | 75 | 7.9 | 63 | 12.1 |
| [other membranes](http://www.arabidopsis.org/servlets/Search?type=gene&action=search&taxon=1&origin=locigoslim&origin_identifier=2944818&slim_name=cytosol) | 81 | 7.0 | 128 | 13.4 | 70 | 13.4 |
| [ribosome](http://www.arabidopsis.org/servlets/Search?type=gene&action=search&taxon=1&origin=locigoslim&origin_identifier=2944818&slim_name=ER) | 80 | 6.9 | 60 | 6.3 | 4 | 0.8 |
| [other cellular components](http://www.arabidopsis.org/servlets/Search?type=gene&action=search&taxon=1&origin=locigoslim&origin_identifier=2944818&slim_name=extracellular) | 60 | 5.2 | 38 | 4.0 | 43 | 8.2 |
| [plasma membrane](http://www.arabidopsis.org/servlets/Search?type=gene&action=search&taxon=1&origin=locigoslim&origin_identifier=2944818&slim_name=Golgi apparatus) | 60 | 5.2 | 42 | 4.4 | 27 | 5.2 |
| [plastid](http://www.arabidopsis.org/servlets/Search?type=gene&action=search&taxon=1&origin=locigoslim&origin_identifier=2944818&slim_name=mitochondria) | 43 | 3.7 | 40 | 4.2 | 38 | 7.3 |
| [cell wall](http://www.arabidopsis.org/servlets/Search?type=gene&action=search&taxon=1&origin=locigoslim&origin_identifier=2944818&slim_name=nucleus) | 39 | 3.4 | 25 | 2.6 | 59 | 11.3 |
| [nucleus](http://www.arabidopsis.org/servlets/Search?type=gene&action=search&taxon=1&origin=locigoslim&origin_identifier=2944818&slim_name=other cellular components) | 36 | 3.1 | 28 | 2.9 | 24 | 4.6 |
| [extracellular](http://www.arabidopsis.org/servlets/Search?type=gene&action=search&taxon=1&origin=locigoslim&origin_identifier=2944818&slim_name=other cytoplasmic components) | 32 | 2.8 | 6 | 0.6 | 22 | 4.2 |
| [mitochondria](http://www.arabidopsis.org/servlets/Search?type=gene&action=search&taxon=1&origin=locigoslim&origin_identifier=2944818&slim_name=other intracellular components) | 18 | 1.6 | 38 | 4.0 | 37 | 7.1 |
| [Golgi apparatus](http://www.arabidopsis.org/servlets/Search?type=gene&action=search&taxon=1&origin=locigoslim&origin_identifier=2944818&slim_name=other membranes) | 15 | 1.3 | 14 | 1.5 | 9 | 1.7 |
| [unknown cellular components](http://www.arabidopsis.org/servlets/Search?type=gene&action=search&taxon=1&origin=locigoslim&origin_identifier=2944818&slim_name=plasma membrane) | 4 | 0.3 | 7 | 0.7 | 2 | 0.4 |
| [ER](http://www.arabidopsis.org/servlets/Search?type=gene&action=search&taxon=1&origin=locigoslim&origin_identifier=2944818&slim_name=plastid) | 0 | 0.0 | 6 | 0.6 | 7 | 1.3 |
| **GO Molecular Function** | **Soluble** | | **SDS** | | **CHAPSO** | |
|  | **Number of proteins** | **% of total** | **Number of proteins** | **% of total** | **Number of proteins** | **% of total** |
| other enzyme activity | [125](http://www.arabidopsis.org/servlets/Search?type=gene&action=search&taxon=1&origin=locigoslim&origin_identifier=2944804&slim_name=cell wall) | 26.4 | 29 | 10.2 | 66 | 22.4 |
| other binding | [88](http://www.arabidopsis.org/servlets/Search?type=gene&action=search&taxon=1&origin=locigoslim&origin_identifier=2944804&slim_name=chloroplast) | 18.6 | 36 | 12.7 | 62 | 21.0 |
| nucleotide binding | [53](http://www.arabidopsis.org/servlets/Search?type=gene&action=search&taxon=1&origin=locigoslim&origin_identifier=2944804&slim_name=cytosol) | 11.2 | 30 | 10.6 | 27 | 9.2 |
| hydrolase activity | [52](http://www.arabidopsis.org/servlets/Search?type=gene&action=search&taxon=1&origin=locigoslim&origin_identifier=2944804&slim_name=ER) | 11.0 | 38 | 13.4 | 44 | 14.9 |
| transferase activity | [39](http://www.arabidopsis.org/servlets/Search?type=gene&action=search&taxon=1&origin=locigoslim&origin_identifier=2944804&slim_name=extracellular) | 8.2 | 19 | 6.7 | 18 | 6.1 |
| protein binding | [38](http://www.arabidopsis.org/servlets/Search?type=gene&action=search&taxon=1&origin=locigoslim&origin_identifier=2944804&slim_name=Golgi apparatus) | 8.0 | 27 | 9.5 | 28 | 9.5 |
| structural molecule activity | [33](http://www.arabidopsis.org/servlets/Search?type=gene&action=search&taxon=1&origin=locigoslim&origin_identifier=2944804&slim_name=mitochondria) | 7.0 | 23 | 8.1 | 10 | 3.4 |
| DNA or RNA binding | [19](http://www.arabidopsis.org/servlets/Search?type=gene&action=search&taxon=1&origin=locigoslim&origin_identifier=2944804&slim_name=nucleus) | 4.0 | 13 | 4.6 | 14 | 4.7 |
| kinase activity | [9](http://www.arabidopsis.org/servlets/Search?type=gene&action=search&taxon=1&origin=locigoslim&origin_identifier=2944804&slim_name=other cellular components) | 1.9 | 7 | 2.5 | 1 | 0.3 |
| other molecular functions | [9](http://www.arabidopsis.org/servlets/Search?type=gene&action=search&taxon=1&origin=locigoslim&origin_identifier=2944804&slim_name=other cytoplasmic components) | 1.9 | 9 | 3.2 | 10 | 3.4 |
| unknown molecular functions | [4](http://www.arabidopsis.org/servlets/Search?type=gene&action=search&taxon=1&origin=locigoslim&origin_identifier=2944804&slim_name=other intracellular components) | 0.8 | 10 | 3.5 | 6 | 2.0 |
| nucleic acid binding | [3](http://www.arabidopsis.org/servlets/Search?type=gene&action=search&taxon=1&origin=locigoslim&origin_identifier=2944804&slim_name=other membranes) | 0.6 | 2 | 0.7 | 3 | 1.0 |
| transcription factor activity | [1](http://www.arabidopsis.org/servlets/Search?type=gene&action=search&taxon=1&origin=locigoslim&origin_identifier=2944804&slim_name=plasma membrane) | 0.2 | 1 | 0.4 | 1 | 0.3 |
| transporter activity | [1](http://www.arabidopsis.org/servlets/Search?type=gene&action=search&taxon=1&origin=locigoslim&origin_identifier=2944804&slim_name=plastid) | 0.2 | 39 | 13.8 | 5 | 1.7 |
| receptor binding or activity | [0](http://www.arabidopsis.org/servlets/Search?type=gene&action=search&taxon=1&origin=locigoslim&origin_identifier=2944804&slim_name=ribosome) | 0.0 | 0 | 0.0 | 0 | 0.0 |
| **GO Biological Process** | **Soluble** | | [**SDS**](http://www.arabidopsis.org/servlets/Search?type=gene&action=search&taxon=1&origin=locigoslim&origin_identifier=2944804&slim_name=developmental processes) | | **CHAPSO** | |
|  | **Number of proteins** | **% of total** | [**Number of proteins**](http://www.arabidopsis.org/servlets/Search?type=gene&action=search&taxon=1&origin=locigoslim&origin_identifier=2944804&slim_name=DNA or RNA metabolism) | **% of total** | **Number of proteins** | **% of total** |
| [other cellular processes](http://www.arabidopsis.org/servlets/Search?type=gene&action=search&taxon=1&origin=locigoslim&origin_identifier=2944818&slim_name=cell wall) | 242 | [27.5](http://www.arabidopsis.org/servlets/Search?type=gene&action=search&taxon=1&origin=locigoslim&origin_identifier=2944783&slim_name=cell organization and biogenesis) | 152 | 28.8 | 141 | 27.0 |
| [other metabolic processes](http://www.arabidopsis.org/servlets/Search?type=gene&action=search&taxon=1&origin=locigoslim&origin_identifier=2944818&slim_name=chloroplast) | 229 | [26.0](http://www.arabidopsis.org/servlets/Search?type=gene&action=search&taxon=1&origin=locigoslim&origin_identifier=2944783&slim_name=developmental processes) | 104 | 19.7 | 126 | 24.1 |
| [response to abiotic or biotic stimulus](http://www.arabidopsis.org/servlets/Search?type=gene&action=search&taxon=1&origin=locigoslim&origin_identifier=2944818&slim_name=cytosol) | 87 | [9.9](http://www.arabidopsis.org/servlets/Search?type=gene&action=search&taxon=1&origin=locigoslim&origin_identifier=2944783&slim_name=DNA or RNA metabolism) | 42 | 8.0 | 50 | 9.6 |
| [response to stress](http://www.arabidopsis.org/servlets/Search?type=gene&action=search&taxon=1&origin=locigoslim&origin_identifier=2944818&slim_name=ER) | 87 | [9.9](http://www.arabidopsis.org/servlets/Search?type=gene&action=search&taxon=1&origin=locigoslim&origin_identifier=2944783&slim_name=electron transport or energy pathways) | 41 | 7.8 | 64 | 12.3 |
| [other biological processes](http://www.arabidopsis.org/servlets/Search?type=gene&action=search&taxon=1&origin=locigoslim&origin_identifier=2944818&slim_name=extracellular) | 74 | [8.4](http://www.arabidopsis.org/servlets/Search?type=gene&action=search&taxon=1&origin=locigoslim&origin_identifier=2944783&slim_name=other biological processes) | 15 | 2.8 | 31 | 5.9 |
| [protein metabolism](http://www.arabidopsis.org/servlets/Search?type=gene&action=search&taxon=1&origin=locigoslim&origin_identifier=2944818&slim_name=Golgi apparatus) | 63 | [7.2](http://www.arabidopsis.org/servlets/Search?type=gene&action=search&taxon=1&origin=locigoslim&origin_identifier=2944783&slim_name=other cellular processes) | 38 | 7.2 | 24 | 4.6 |
| [developmental processes](http://www.arabidopsis.org/servlets/Search?type=gene&action=search&taxon=1&origin=locigoslim&origin_identifier=2944818&slim_name=mitochondria) | 37 | [4.2](http://www.arabidopsis.org/servlets/Search?type=gene&action=search&taxon=1&origin=locigoslim&origin_identifier=2944783&slim_name=other metabolic processes) | 20 | 3.8 | 14 | 2.7 |
| [cell organization and biogenesis](http://www.arabidopsis.org/servlets/Search?type=gene&action=search&taxon=1&origin=locigoslim&origin_identifier=2944818&slim_name=nucleus) | 31 | [3.5](http://www.arabidopsis.org/servlets/Search?type=gene&action=search&taxon=1&origin=locigoslim&origin_identifier=2944783&slim_name=protein metabolism) | 26 | 4.9 | 31 | 5.9 |
| [transport](http://www.arabidopsis.org/servlets/Search?type=gene&action=search&taxon=1&origin=locigoslim&origin_identifier=2944818&slim_name=other cellular components) | 10 | [1.1](http://www.arabidopsis.org/servlets/Search?type=gene&action=search&taxon=1&origin=locigoslim&origin_identifier=2944783&slim_name=response to abiotic or biotic stimulus) | 52 | 9.8 | 12 | 2.3 |
| [electron transport or energy pathways](http://www.arabidopsis.org/servlets/Search?type=gene&action=search&taxon=1&origin=locigoslim&origin_identifier=2944818&slim_name=other cytoplasmic components) | 9 | [1.0](http://www.arabidopsis.org/servlets/Search?type=gene&action=search&taxon=1&origin=locigoslim&origin_identifier=2944783&slim_name=response to stress) | 12 | 2.3 | 16 | 3.1 |
| [signal transduction](http://www.arabidopsis.org/servlets/Search?type=gene&action=search&taxon=1&origin=locigoslim&origin_identifier=2944818&slim_name=other intracellular components) | 4 | [0.5](http://www.arabidopsis.org/servlets/Search?type=gene&action=search&taxon=1&origin=locigoslim&origin_identifier=2944783&slim_name=signal transduction) | 8 | 1.5 | 2 | 0.4 |
| [DNA or RNA metabolism](http://www.arabidopsis.org/servlets/Search?type=gene&action=search&taxon=1&origin=locigoslim&origin_identifier=2944818&slim_name=other membranes) | 3 | [0.3](http://www.arabidopsis.org/servlets/Search?type=gene&action=search&taxon=1&origin=locigoslim&origin_identifier=2944783&slim_name=transcription,DNA-dependent) | 7 | 1.3 | 1 | 0.2 |
| [unknown biological processes](http://www.arabidopsis.org/servlets/Search?type=gene&action=search&taxon=1&origin=locigoslim&origin_identifier=2944818&slim_name=plasma membrane) | 3 | [0.3](http://www.arabidopsis.org/servlets/Search?type=gene&action=search&taxon=1&origin=locigoslim&origin_identifier=2944783&slim_name=transport) | 10 | 1.9 | 8 | 1.5 |
| [transcription,DNA-dependent](http://www.arabidopsis.org/servlets/Search?type=gene&action=search&taxon=1&origin=locigoslim&origin_identifier=2944818&slim_name=plastid) | 2 | [0.2](http://www.arabidopsis.org/servlets/Search?type=gene&action=search&taxon=1&origin=locigoslim&origin_identifier=2944783&slim_name=unknown biological processes) | 1 | 0.2 | 2 | 0.4 |
