# Supplementary material for: The broccoli (Brassica oleracea) phloem tissue proteome
Source: BMC Genomics. 2013 Nov 7;14:764. doi: 10.1186/1471-2164-14-764 (PMC3833381; doi:10.1186/1471-2164-14-764)
Supplement: Additional file 6: Figure S1 — Semi-quantitative RT-PCR analysis of gene expression in B. oleracea phloem-enriched strands (P) and non-phloem containing control tissue (NP). [file 1471-2164-14-764-S6.pptx]

## Slide 1
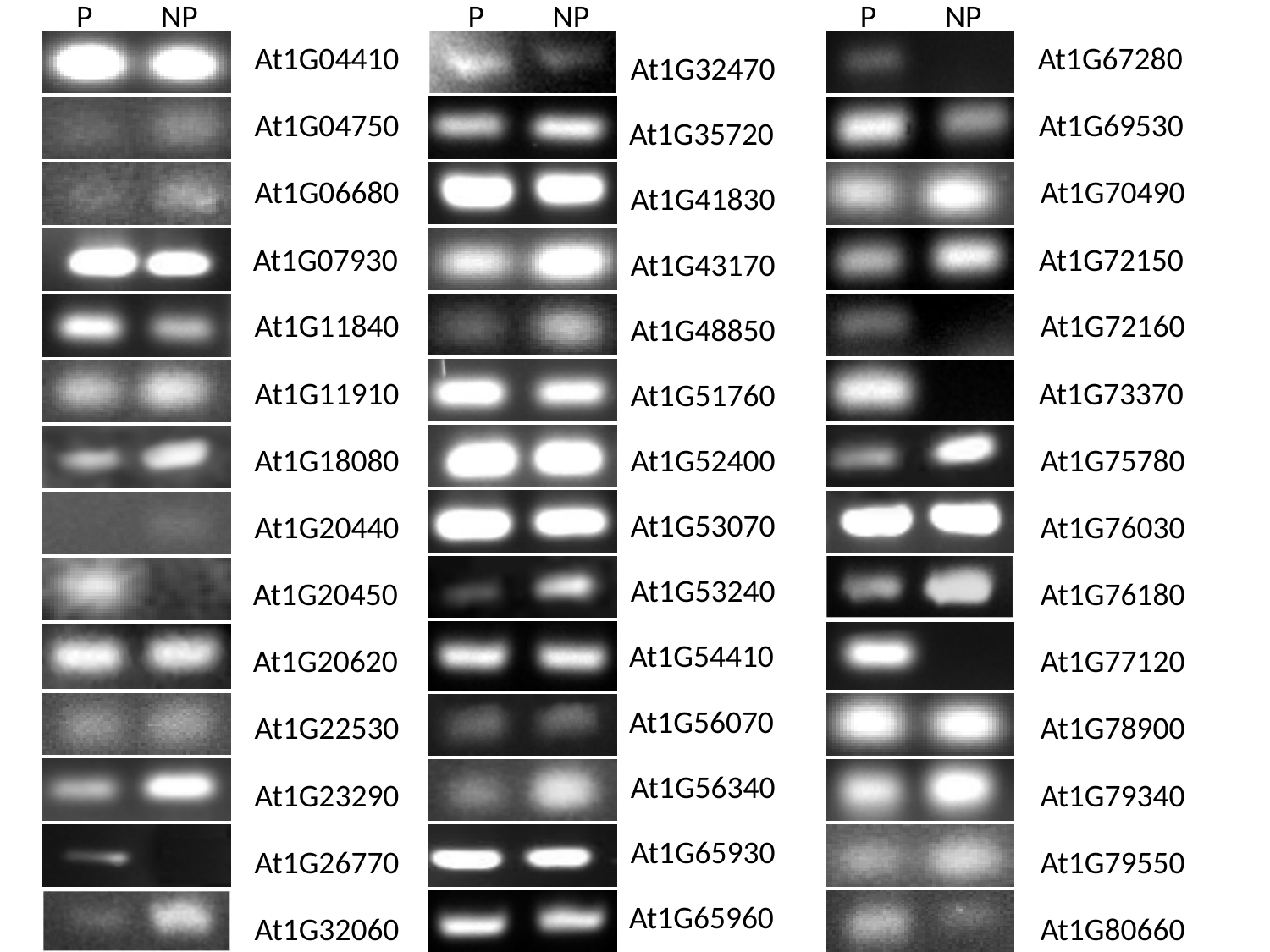

P
NP
P
NP
P
NP
At1G04410
At1G67280
At1G32470
At1G04750
At1G69530
At1G35720
At1G06680
At1G70490
At1G41830
At1G07930
At1G72150
At1G43170
At1G11840
At1G72160
At1G48850
At1G11910
At1G73370
At1G51760
At1G18080
At1G75780
At1G52400
At1G53070
At1G20440
At1G76030
At1G53240
At1G20450
At1G76180
At1G54410
At1G20620
At1G77120
At1G56070
At1G22530
At1G78900
At1G56340
At1G23290
At1G79340
At1G65930
At1G26770
At1G79550
At1G65960
At1G32060
At1G80660

## Slide 2
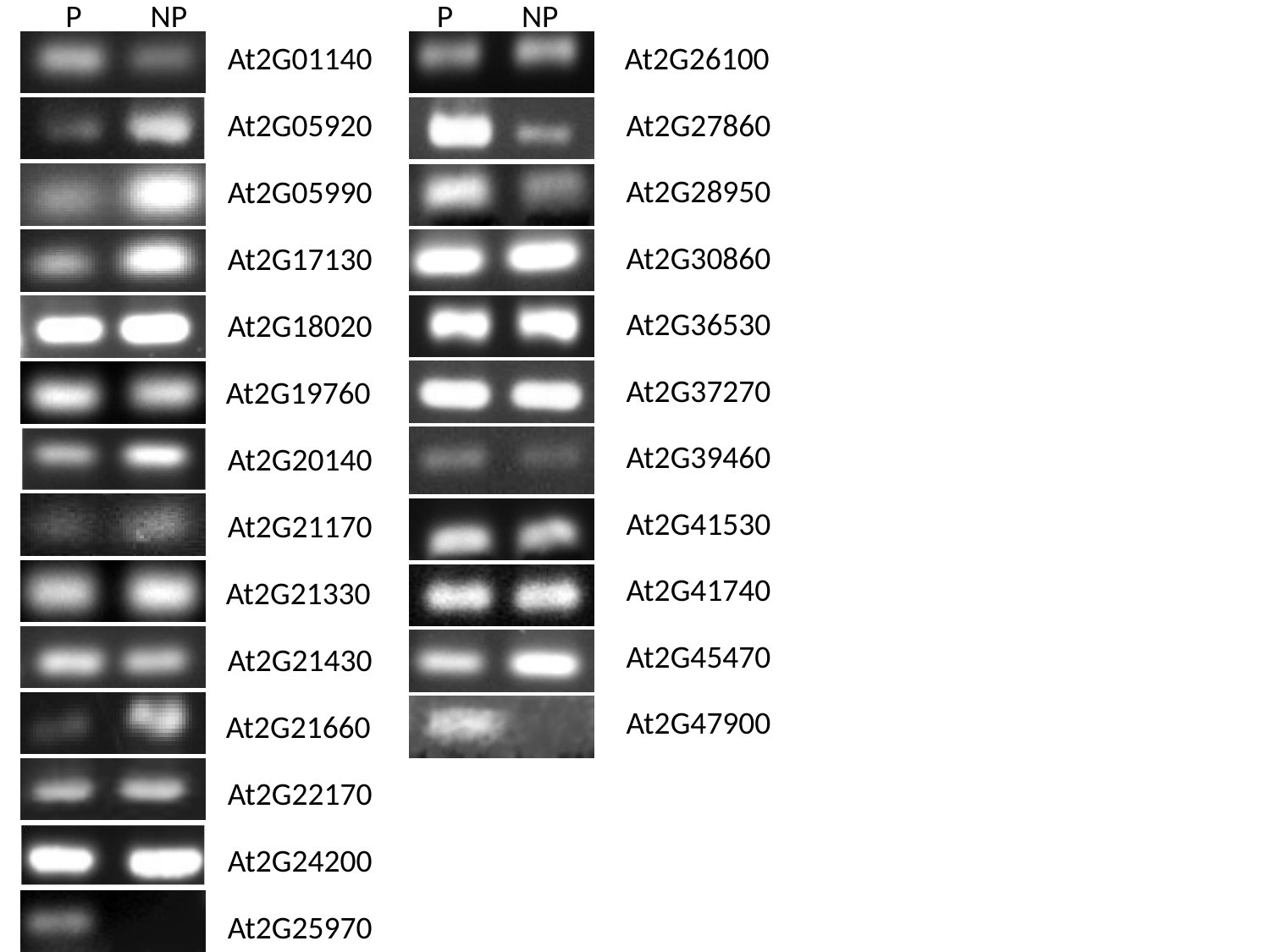

P
NP
P
NP
At2G01140
At2G26100
At2G27860
At2G05920
At2G28950
At2G05990
At2G30860
At2G17130
At2G36530
At2G18020
At2G37270
At2G19760
At2G39460
At2G20140
At2G41530
At2G21170
At2G41740
At2G21330
At2G45470
At2G21430
At2G47900
At2G21660
At2G22170
At2G24200
At2G25970

## Slide 3
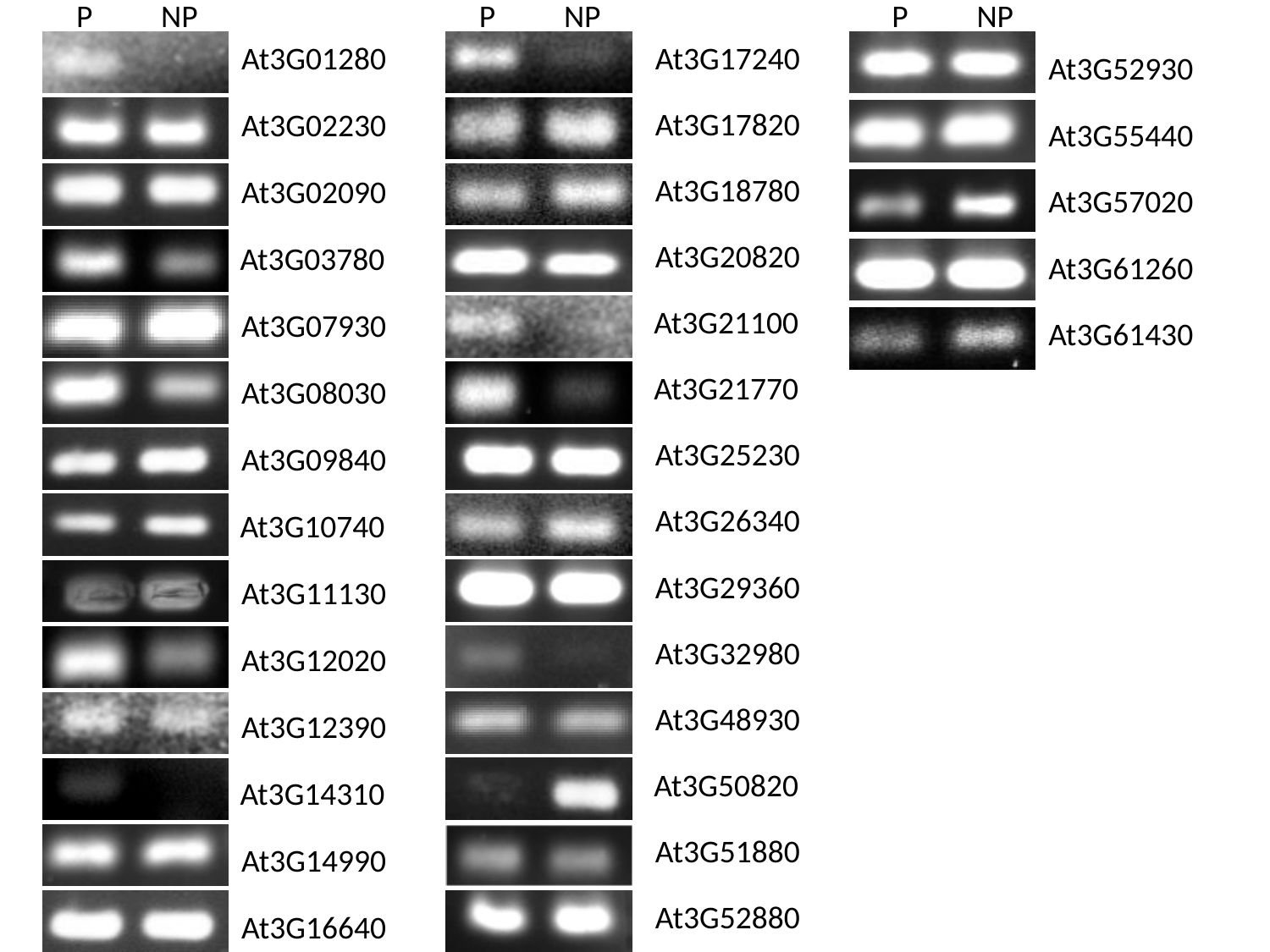

P
NP
P
NP
P
NP
At3G01280
At3G17240
At3G52930
At3G17820
At3G02230
At3G55440
At3G18780
At3G02090
At3G57020
At3G20820
At3G03780
At3G61260
At3G21100
At3G07930
At3G61430
At3G21770
At3G08030
At3G25230
At3G09840
At3G26340
At3G10740
At3G29360
At3G11130
At3G32980
At3G12020
At3G48930
At3G12390
At3G50820
At3G14310
At3G51880
At3G14990
At3G52880
At3G16640

## Slide 4
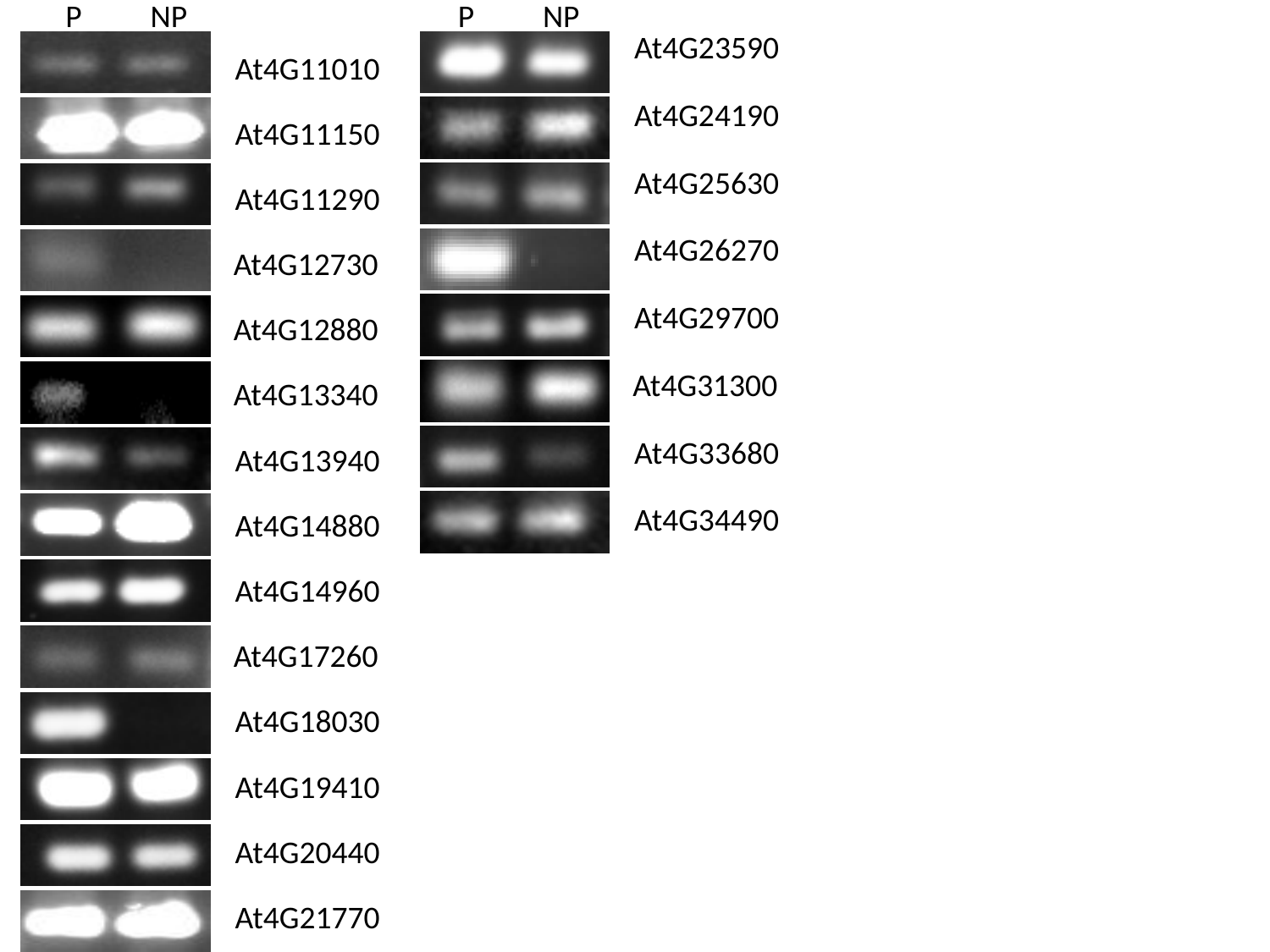

P
NP
P
NP
At4G23590
At4G11010
At4G24190
At4G11150
At4G25630
At4G11290
At4G26270
At4G12730
At4G29700
At4G12880
At4G31300
At4G13340
At4G33680
At4G13940
At4G34490
At4G14880
At4G14960
At4G17260
At4G18030
At4G19410
At4G20440
At4G21770

## Slide 5
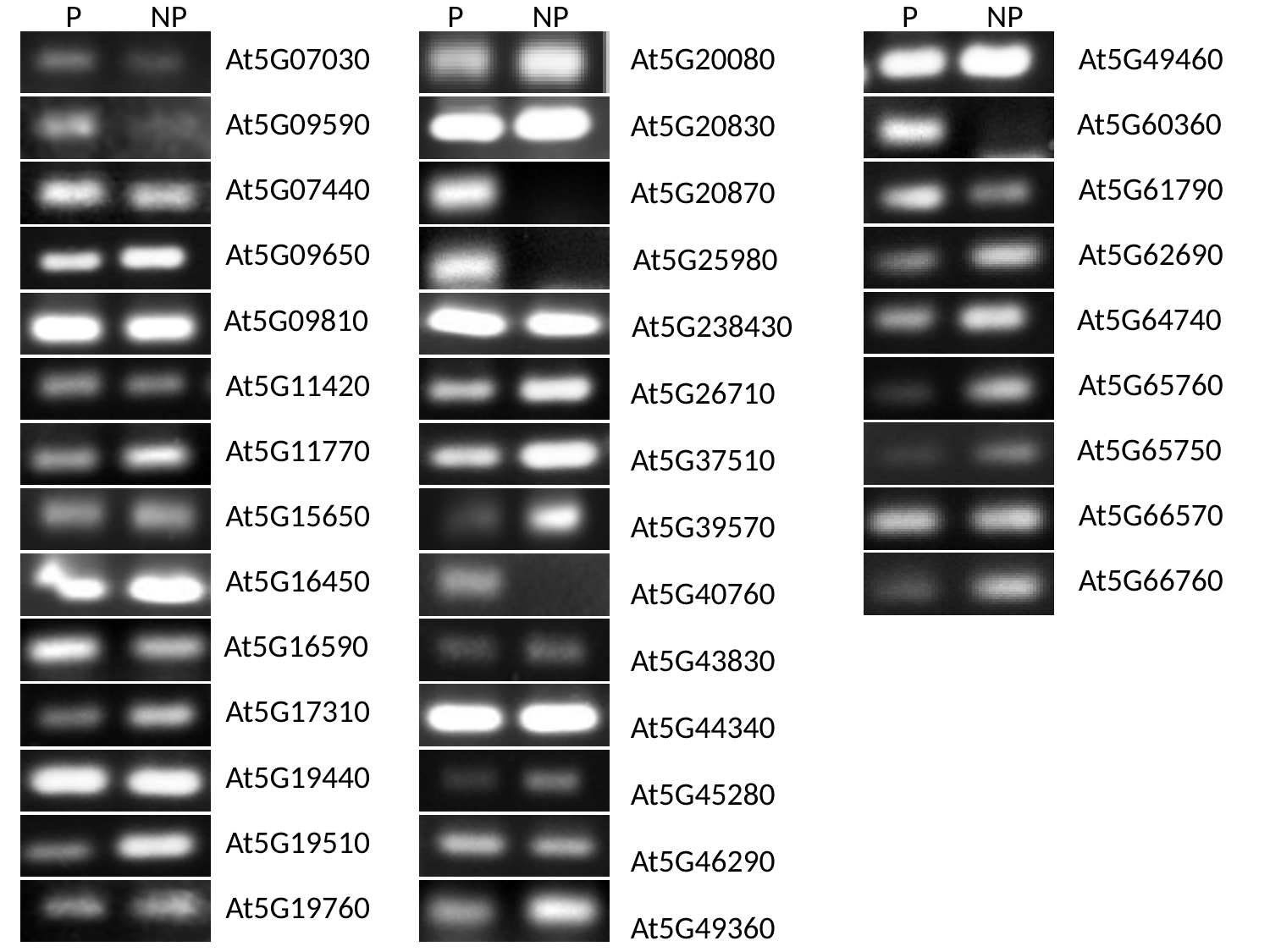

P
NP
P
NP
P
NP
At5G07030
At5G20080
At5G49460
At5G60360
At5G09590
At5G20830
At5G61790
At5G07440
At5G20870
At5G62690
At5G09650
At5G25980
At5G64740
At5G09810
At5G238430
At5G65760
At5G11420
At5G26710
At5G65750
At5G11770
At5G37510
At5G66570
At5G15650
At5G39570
At5G66760
At5G16450
At5G40760
At5G16590
At5G43830
At5G17310
At5G44340
At5G19440
At5G45280
At5G19510
At5G46290
At5G19760
At5G49360
